# Small molecule-mediated allosteric activation of the base excision repair enzyme 8-oxoguanine DNA glycosylase and its impact on mitochondrial function

**DOI:** 10.1038/s41598-022-18878-2

**Published:** 2022-08-29

**Authors:** Gaochao Tian, Steven R. Katchur, Yong Jiang, Jacques Briand, Michael Schaber, Constantine Kreatsoulas, Benjamin Schwartz, Sara Thrall, Alicia M. Davis, Sam Duvall, Brett A. Kaufman, William L. Rumsey

**Affiliations:** 1 GSK, Collegeville, PA 19426 USA; 2 Division of Cardiology, Department of Medicine, Center for Metabolism and Mitochondrial Medicine, University of Pittsburgh, Pittsburgh, PA 15261 USA; 3 Heart, Lung, and Blood Vascular Medicine Institute, University of Pittsburgh, Pittsburgh, PA 15261 USA

**Keywords:** Enzymes, Biochemistry, DNA-binding proteins, Stress signalling, Cell biology, Cellular imaging, Mechanisms of disease, Organelles

## Abstract

8-Oxoguanine DNA glycosylase (OGG1) initiates base excision repair of the oxidative DNA damage product 8-oxoguanine. OGG1 is bifunctional; catalyzing glycosyl bond cleavage, followed by phosphodiester backbone incision via a β-elimination apurinic lyase reaction. The product from the glycosylase reaction, 8-oxoguanine, and its analogues, 8-bromoguanine and 8-aminoguanine, trigger the rate-limiting AP lyase reaction. The precise activation mechanism remains unclear. The product-assisted catalysis hypothesis suggests that 8-oxoguanine and analogues bind at the product recognition (PR) pocket to enhance strand cleavage as catalytic bases. Alternatively, they may allosterically activate OGG1 by binding outside of the PR pocket to induce an active-site conformational change to accelerate apurinic lyase. Herein, steady-state kinetic analyses demonstrated random binding of substrate and activator. 9-Deazaguanine, which can’t function as a substrate-competent base, activated OGG1, albeit with a lower E_max_ value than 8-bromoguanine and 8-aminoguanine. Random compound screening identified small molecules with E_max_ values similar to 8-bromoguanine. Paraquat-induced mitochondrial dysfunction was attenuated by several small molecule OGG1 activators; benefits included enhanced mitochondrial membrane and DNA integrity, less cytochrome *c* translocation, ATP preservation, and mitochondrial membrane dynamics. Our results support an allosteric mechanism of OGG1 and not product-assisted catalysis. OGG1 small molecule activators may improve mitochondrial function in oxidative stress-related diseases.

## Introduction

Genomic DNA, particularly mitochondrial (mt), is prone to oxidative attack and modification by reactive oxygen species (ROS). Endogenous ROS arise primarily as by-products of mitochondrial oxidative phosphorylation or from exogenous exposure to chemicals and physical agents such as ionizing radiation^1^. Among various oxidative DNA products, 8-oxoguanine (8-oxo-dG, Supplementary Table S1) stands out as one of the most abundant in the genome^1–3^. Replication of this damaged base can be highly mutagenic by converting a normal G-C base pair into an 8-oxo-dG-A mismatch, thereby causing a G to T transversion after two rounds of DNA replication^4–6^. Accumulation of 8-oxo-dG has been causally associated (through a yet to be determined mechanism) with several age-related diseases including cancer, neurodegeneration, chronic obstructive pulmonary disease, diabetes, and heart failure^7–13^.

To counteract these potentially mutagenic consequences, cells primarily use base excision repair (BER), which is a complex multi-enzymatic process that is accomplished through the coordinated action of a DNA glycosylase, AP endonuclease, polymerase-β (or polymerase-γ in the mitochondria), as well as a DNA ligase^3,14–16^. 8-Oxoguanine DNA glycosylase (OGG1) is a bifunctional enzyme that catalyzes both a glycosylase and an AP lyase reaction. OGG1 performs the first, and perhaps also the rate-limiting step, in the damage control pathway involving 8-oxo-dG^17^. Several studies have demonstrated that the overexpression of OGG1, particularly within the mitochondrial compartment, confers cellular protection in assorted cellular and animal models; for examples, see^18–21^. These findings support the view that accelerating OGG1 activity, especially within mitochondria, may be beneficial to mitochondrial function by minimizing the accumulation of mutagenic and cytotoxic 8-oxo-dG in DNA during periods of oxidative stress.

**Figure 1:**
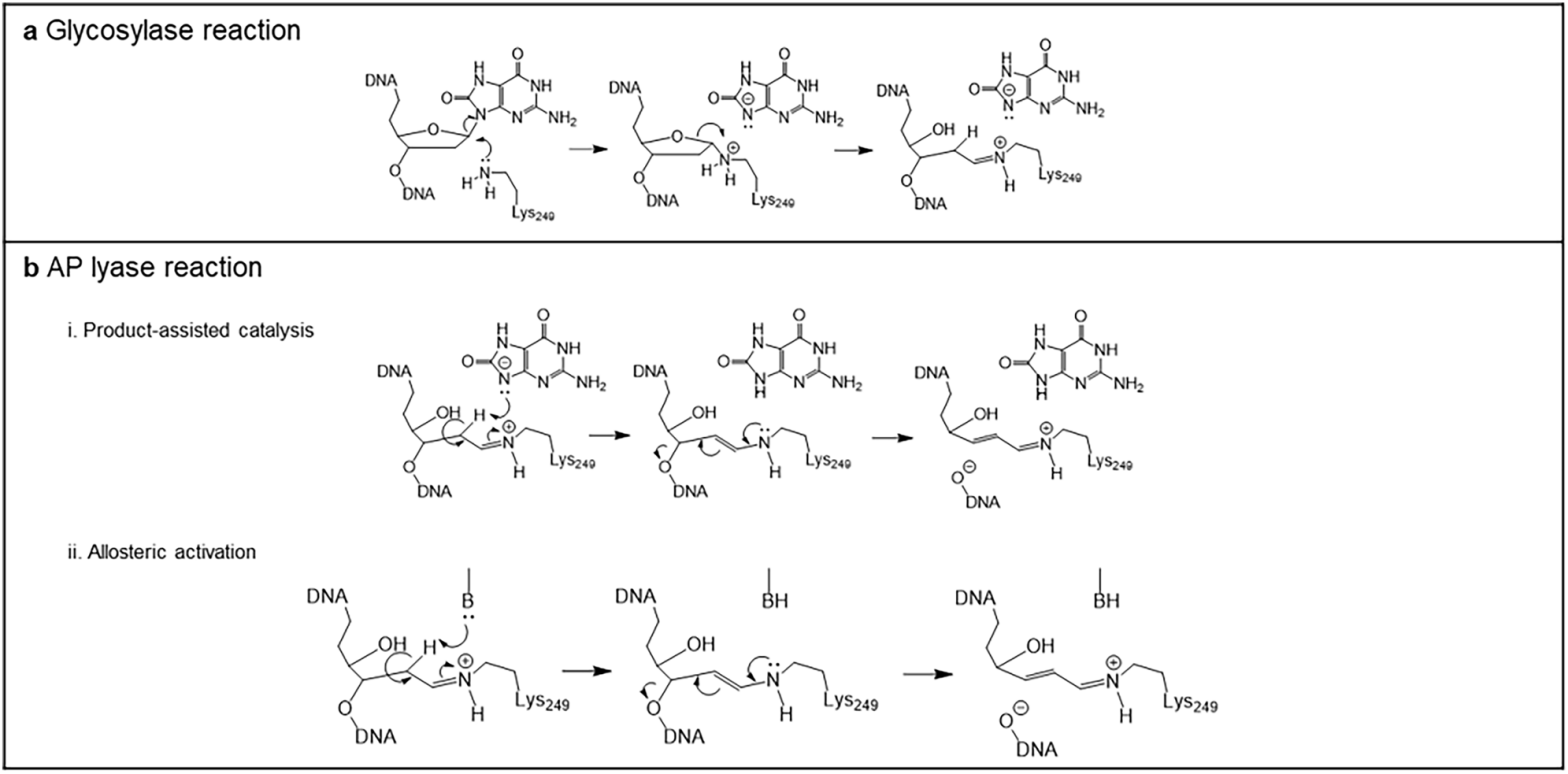
OGG1 catalytic mechanism. The panels describe the structural interactions between DNA containing 8-oxo-dG and OGG1 represented by the lys_249_ amino acid. In (**a**), the glycosylase reaction is displayed and in (**b**) the AP lyase one with the projected re-arrangements resulting from product assisted catalysis (i) or allosteric modification (ii).

Once OGG1 recognizes an 8-oxo-dG lesion within DNA, it efficiently removes 8-oxo-dG from the damage site through its glycosylase activity via a nucleophilic attack on C1′ of the aberrant nucleotide by the active site residue Lys249^18,22^. This attack leads to the formation of a Schiff base adduct between the ε-amino group of Lys249 and C1′ of the abasic product (Fig. 1a). Residue Lys249, as well as Asp268, are important for catalysis and conformational adjustment of the damaged nucleotide in the active site^23^. The rapid nucleobase removal is followed by a much slower AP lyase activity initiated through C2′-proton abstraction that results in β-elimination of the 3′-phosphate group and concurrent DNA strand cleavage.

Although the glycosylase reaction is relatively well understood, the mechanism that underpins AP lyase activity, particularly β-elimination, i.e., C2′ proton abstraction, is less clear. It has been proposed^24^ that β-elimination is achieved through an unprecedented mechanism called “product-assisted catalysis”, in which the 8-oxoGua (glycosylase) product is retained at the product recognition pocket. The N9-atom of the glycosylase product then serves as the catalytic base for C2′-proton abstraction, which is the trigger step for the AP lyase reaction (Fig. 1b i). Consistent with this scenario, in the co-crystal structure of OGG1 and a lesion-containing DNA substrate, the liberated 8-oxoGua is seen sequestered at the active site with the N9-atom located near C2′ at a distance seemingly competent for 8-oxoGua to abstract a proton from C2′^24^. The 8-oxoGua analogs, 8-bromoguanine (8-bromoGua) and 8-aminoguanine (8-aminoGua, Supplementary Table S1) were also reported to activate AP lyase activity, accelerating this less efficient reaction by > 75- and > 35-fold and with a substrate turnover of 89% and 72%, respectively^24^. This observed difference in activation levels by these nucleobases was attributed to electron effects on catalysis, supporting the product-assisted catalysis hypothesis. Subsequently, Kuznetsov and coworkers^25^ suggested that: 1) binding of 8-bromoGua in the catalytic pocket would competitively inhibit enzyme activity; 2) product-assisted catalysis may be interesting mechanistically but may not be relevant in vivo and 3) 8-bromoGua might be an allosteric activator of AP lyase activity although structural support for the latter was lacking. However, binding of free 8-oxoGua at a non-substrate site on OGG1 has been shown to occur with sub-nM affinity and produced a conformational change as measured by intrinsic OGG1 tryptophan fluorescence^26^. Thus, binding of an activator with free enzyme at the catalytic site would prevent binding of substrate and would result in enzyme inhibition rather than activation. On the other hand, direct binding to free enzyme is consistent with allosteric activation in which 8-oxoGua or its analogues bind to a site outside of the nucleobase recognition pocket to induce a conformational change in the active site. This would move an otherwise misplaced, enzyme-derived base group into a proximal position for C2′-proton abstraction (Fig. 1b ii).

In the current investigation, we present results from a mechanistic assessment of enhanced OGG1 catalytic activity coupled with molecular modeling of potential ligand binding sites and biophysical measures utilizing OGG1 activators that were identified from random compound screening. Moreover, some of these compounds were evaluated in quantitative cellular imaging studies of mitochondrial function in response to oxidative stress. The present data are at odds with the hypothesis of product-assisted catalysis and support an allosteric activation mechanism by product analogs and non-nucleobase small molecule activators. The functional studies herein demonstrate several mitochondrial benefits of OGG1 activation by small molecules in agreement with our earlier work^27^ and are consistent with results previously obtained by OGG1 overexpression in the mitochondria^18–21^.

## Results

### Development of a fluorescent steady-state kinetic assay for initial rate analysis

To understand the mechanism of human OGG1 activation, a detailed initial rate analysis was carried out. A continuous kinetic assay that followed reaction progress curves was desirable for this purpose using molecular beacon technology^28^ with minor modification: an 8-oxo-dG-containing hairpin DNA substrate (Substrate A) with a fluorescent quench pair of 5′-FAM and 3′-DAB (Supplementary Fig. S1 online). The substrate from the earlier design contained five base pairs 5′ to 8-oxo-dG in the stem of the hairpin DNA substrate. Upon strand cleavage by OGG1, the FAM-containing cleavage product dissociates from the rest of the molecular beacon substrate, thereby releasing the quench effect of 3′-DAB and increasing fluorescence at 522 nm upon excitation at 485 nm. The evolving fluorescence emitted from the early design substrate at 100 nM in the presence of 250 μM 8-bromoGua was linear for a short time (~ 10 min), but then leveled off at a less than stoichiometric substrate turnover (Supplementary Fig. S1). This preliminary result suggested that the cleaved pentameric DNA oligodeoxynucleotide was not completely dissociated from the larger cleavage product at reaction equilibrium. In the current design, the number of nucleotide pairs 5′ to the 8-oxo-dG was trimmed from five to three (Substrate A, Supplementary Fig. S1) to reduce the thermodynamic energy controlling dissociation of the FAM-containing cleavage product. The time course of the OGG1 reaction using 100 nM Substrate A in the presence of 200 μM 8-bromoGua was linear up to ~ 40 min, allowing accurate measurements of initial rates using this substrate (Supplementary Fig. S1).

### Initial rate analysis indicates random kinetics for OGG1 activation by 8-bromoGua

**Figure 2:**
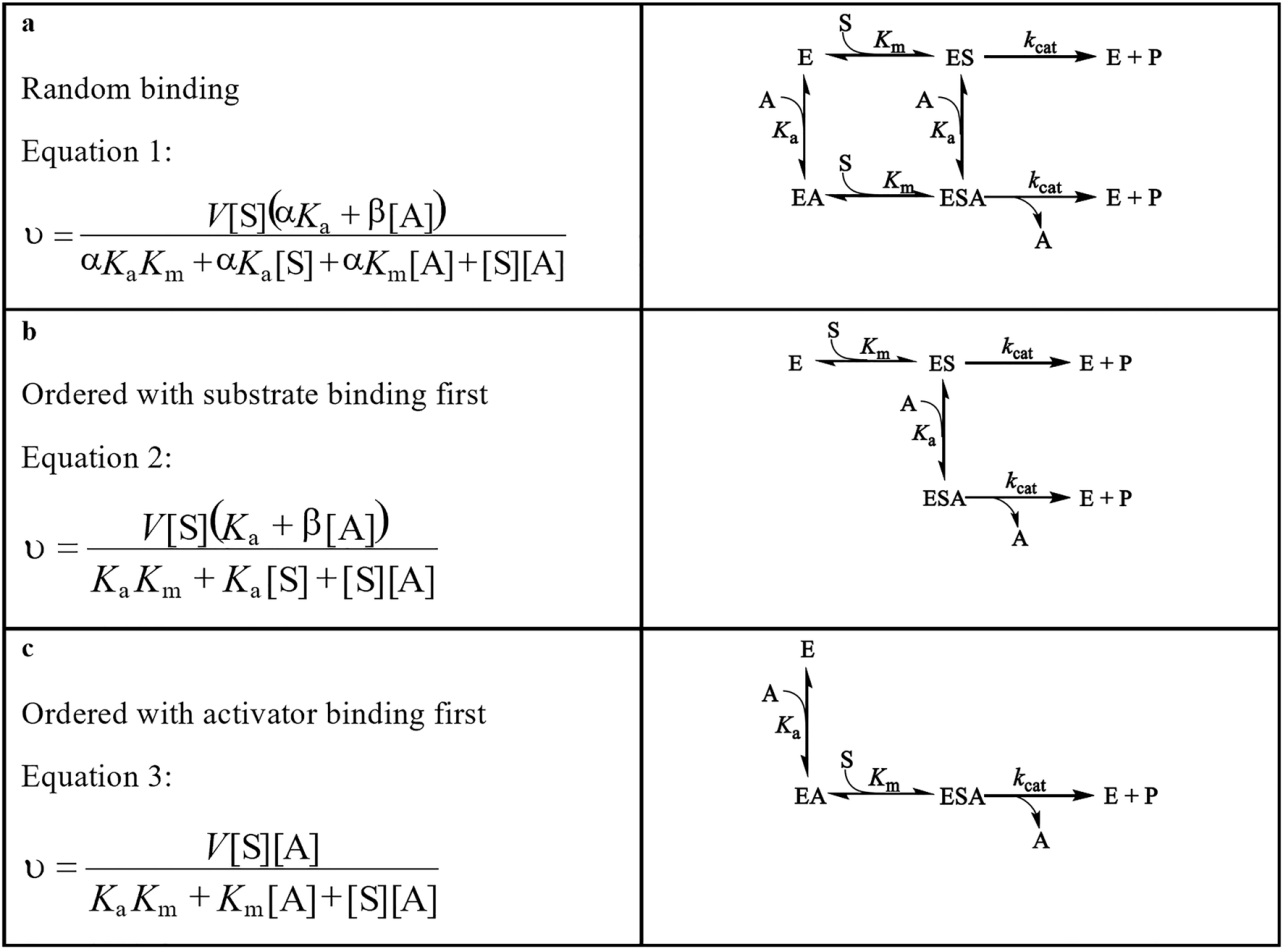
Kinetic mechanisms of enzyme activation. In these kinetic schemes, the symbols represent: E, enzyme, S, substrate, A, activator, K_m_, Michaelis-Menton constant, K_a_, activation constant, α, the coefficient of interaction between activator and substrate, and β, factor of k_*cat*_ activation. Equations that describe these mechanisms of random binding, ordered with substrate binding first, and ordered with activator binding first are also provided where V is the maximal initial rate (k_*cat*_Et, in which Et is the total enzyme concentration). Further details are found in the Supplementary Table S2.

Having established a continuous kinetic fluorescent assay, we examined the steady state kinetic mechanism of OGG1 activation by 8-bromoGua. In general, three distinct kinetic mechanisms regarding the order of substrate and activator binding are possible (Fig. 2). Crystallographic evidence indicates that upon binding of an 8-oxo-dG-containing DNA oligodeoxynucleotide to OGG1, the aberrant nucleobase is extruded from the DNA helix and inserted into an 8-oxo-dG recognition pocket on the enzyme^29^. This transition is a prerequisite step to cleavage of the glycosidic bond of the damaged base. Product-assisted catalysis (Fig. 1b i) necessitates an ordered mechanism whereby the substrate binds first, and the activator binds only after the substrate is bound with 8-oxo-dG having been cleaved and the product recognition pocket vacated. If substrate binding occurs after association of the activator to the free enzyme at the product recognition pocket, the flipping of the aberrant nucleobase into this pocket for cleavage would be blocked. Consequently, inhibition rather than activation of the overall activity of OGG1 would result. On the other hand, allosteric activation (Fig. 1b ii) could operate by several mechanisms. For example, for a random binding mechanism (Fig. 2a), an allosteric activator could enhance the OGG1 activity by increasing the affinity of the enzyme for its substrate (α < 1) under non-saturation conditions or by increasing *k*_cat_ (β > 1), or by a combination of the two (α < 1 and β > 1). For an ordered mechanism with substrate binding first (Fig. 2b), the activation is only realized by increasing *k*_cat_ (β > 1). Alternately, in an ordered mechanism with activator bound first (Fig. 2c), it binds only to the free enzyme which is necessary for substrate binding and enzyme activity. Because OGG1 is known to have a basal activity in the absence of an activator^22^, the ordered mechanism with activator binding first cannot account for OGG1 activation. Therefore, only the random mechanism and the ordered with substrate bound first will be considered henceforth.

**Figure 3:**
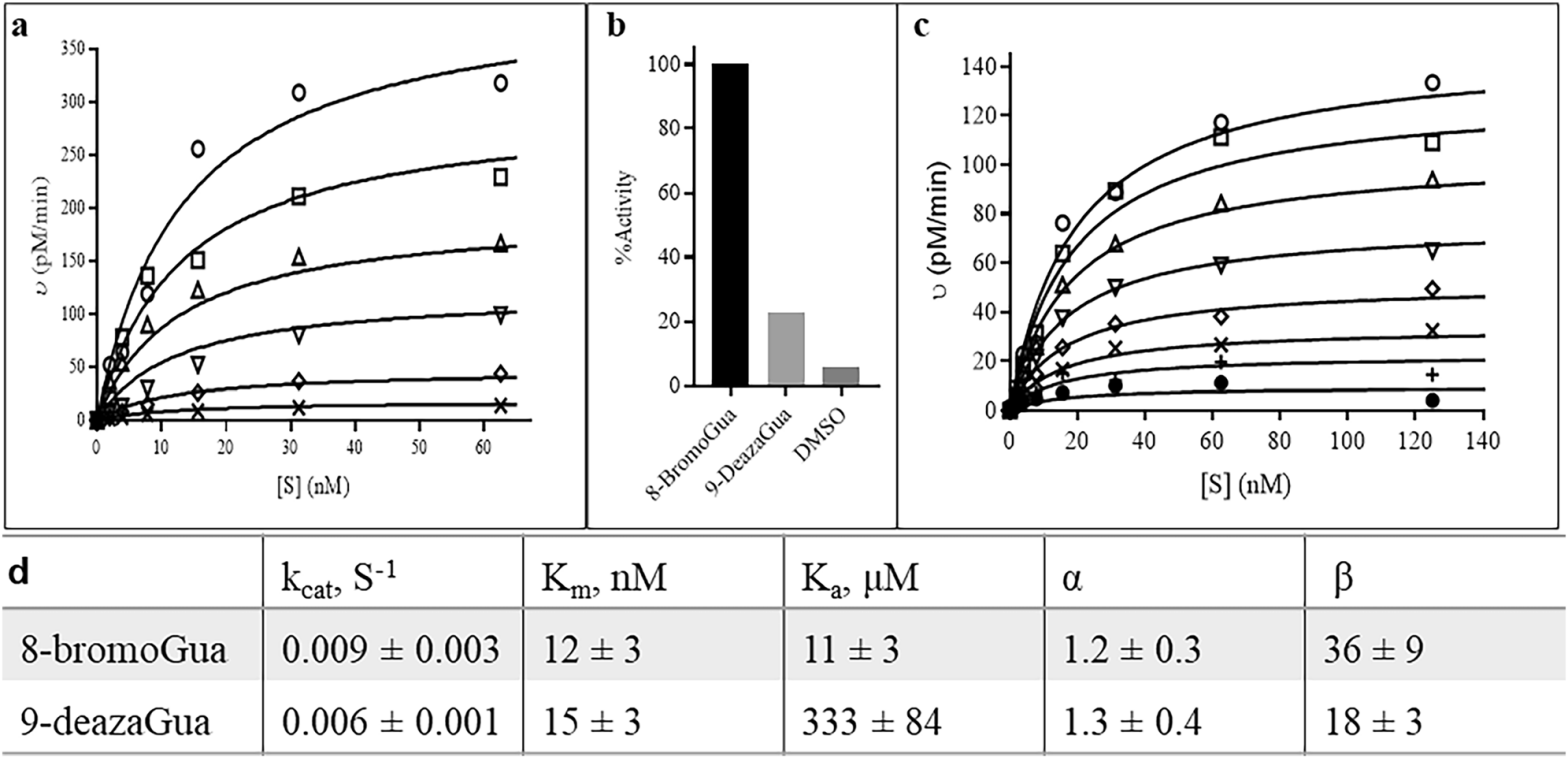
Activation of OGG1 by 8-bromoGua and 9-deazaGua. The term [S] refers to substrate A displayed in Supplementary Fig. S1. Values were obtained in duplicate from an independent experiment by varying substrate concentration over time. In (**a**) OGG1 (at 10 nM) initial rates were measured as a function of substrate A concentration listed on the x-axis in the presence of 8-bromoGua at 0.0 (×), 0.63 (♢), 2.5 (▽), 5 (△), 10 (◻), and 20 (○) μM. Kinetic parameters from analyzing the data using Eq. (2) were used to calculate the theoretical values (solid lines). (**b**) Bar plot of OGG1 activity in the presence of 200 μM 8-bromoGua and 9-deazaGua. Data were normalized to OGG1 activity in the presence of 200 μM 8-bromoGua. (**c**) Similarly, to panel (**a**), plots of OGG1 (at 10 nM) initial rates as a function of substrate A concentration (x-axis) in the presence of 9-deazaGua at 0.0 (●), 39 (+), 78 (×), 156 (♢), 313 (▽), 625 (△), 1250 (◻), and 2500 (○) μM. (**d**) Kinetic parameters from analyzing the data using Eq. (2) (Supplementary Table 2) were used to calculate the theoretical values (solid lines). Standard deviation of the fit of the lines provides the error terms.

To perform the initial rate analysis, the concentrations of both substrate and 8-bromoGua were varied in the fluorescent kinetic assay and the corresponding initial rates calculated using data points from the linear portion of the incision reactions revealed by the progress curves. The initial rates were then analyzed via least squares analysis according to Eqs. (1 and 2) (Fig. 2a,b), which describe the mechanisms of random binding and ordered with substrate binding first, respectively. The random kinetic mechanism fits best to the experimental results, with the calculated α value being close to unity (1.2 ± 0.3 μM, Fig. 3d). Figure 3a depicts initial rates as a function of substrate concentration in the presence of different fixed concentrations of activator as well as corresponding theoretical lines that were calculated using the parameters derived from the least squares analysis according to the random kinetic mechanism. The *K*_a_ value of 12 ± 3 μM (Fig. 3d) agrees well with values reported in the literature from a dose–response analysis at a single fixed substrate concentration i.e., 9 μM^24^. The β value, which determines the extent to which the maximum rate of AP lyase is enhanced in the presence of an activator, was 36 ± 9 μM (Fig. 3d). Because binding of activator to free enzyme would inhibit, rather than activate OGG1 as described above, these results which were derived using Eq. (1) (Fig. 2a, random binding) where an activator can bind to the free enzyme as well as the ES complex with a similar affinity (α = 1.2 ± 0.3 μM, Fig. 3d), were the first strong piece of evidence to suggest that OGG1 activation by product or product-analogs may not occur through product-assisted catalysis.

### 9-Deazaguanine activates β-elimination by OGG1

The N9-atom is absolutely required for base catalysis. As such, a 9-deazaGua analog (Supplementary Table S1 online) would not be expected to activate OGG1 according to product-assisted catalysis. Thus, we next tested the impact of 9-deazaGua on OGG1 activity in the fluorescent kinetic assay. First, the initial rate of OGG1 was evaluated in the presence of 200 μM 9-deazaGua at a substrate concentration (80 nM) which was close to enzyme saturation (*K*_m_ = 12–15 nM, Fig. 3d). Under the same experimental conditions, this activity was ~ 25% of the initial rate of OGG1 in the presence of 200 μM 8-bromoGua (Fig. 3b). Having demonstrated OGG1 activation by 9-deazaGua relative to vehicle alone, a full kinetic initial rate analysis of OGG1 with varying concentrations of both Substrate A and 9-deazaGua was performed. The initial rates from the latter analysis were best fit with the random mechanism (Eq. 1) by least-squares analysis. Figure 3c depicts the fit results and Fig. 3d summarizes the kinetic parameters obtained from this analysis. The *k*_cat_ value of 0.006 ± 1 s^-1^ obtained from this analysis is in good agreement with the 0.009 ± 1 s^-1^ value obtained from the initial rate analysis of OGG1 in the presence of 8-bromoGua (Fig. 3d). Although 9-deazaGua displays a relatively poor affinity for OGG1 (*K*_a_ = 333 ± 84 μM), the β value, which measures the fold of *k*_cat_ activation, is 18 ± 3 which is 50% of the catalytic activity of OGG1 in the presence of 8-bromoGua (36 ± 9, Fig. 3d). The purity of the 9-deazaGua sample used in this analysis was determined by LCMS to be > 99.5%, thus the observed activation was not due to contamination. Given that 9-deazaGua lacks a catalytic base, this result demonstrated that product-assisted catalysis is not the principal mechanism for the β-elimination step catalyzed by OGG1.

### Random screening identifies several small molecule activators of OGG1

The results described above support an observed random kinetic mechanism for allosteric activation of OGG1 by 9-deazaGua and dispute product-assisted catalysis. Thus, if the activation of OGG1 is indeed allosteric, small molecules that are structurally distinct from product analogs could be discovered as OGG1 activators. To evaluate this possibility, a random screening of small molecules in the GSK compound collection was carried out using the fluorescent kinetic assay described above. Interestingly, several small molecules with diverse structures (examples include compounds **I-XIII**; Supplementary Table S3) were detected that robustly activated OGG1. The *E*_max_ values of these compounds (range = 77–118%, EC_50_ values = 0.35–6.4 μM) were similar to the *E*_max_ value obtained for 8-bromoGua, which served as the positive control. These data indicated that the activation efficiency of these compounds, which were structurally distinct from product analogs, was as great or greater than that of 8-bromoGua. It is difficult to reconcile this outcome by product-assisted catalysis. Rather the results can be easily explained by allosteric activation, in which activation is not achieved through direct involvement of activator in catalysis, but by a conformational change at the active site upon binding of activator to an allosteric binding pocket. The latter step reorients the enzyme-derived catalytic base group for C2′-proton abstraction (Fig. 1b ii).

### ^19^F NMR line-broadening analysis demonstrates binding of Compound VI to free OGG1

**Figure 4:**
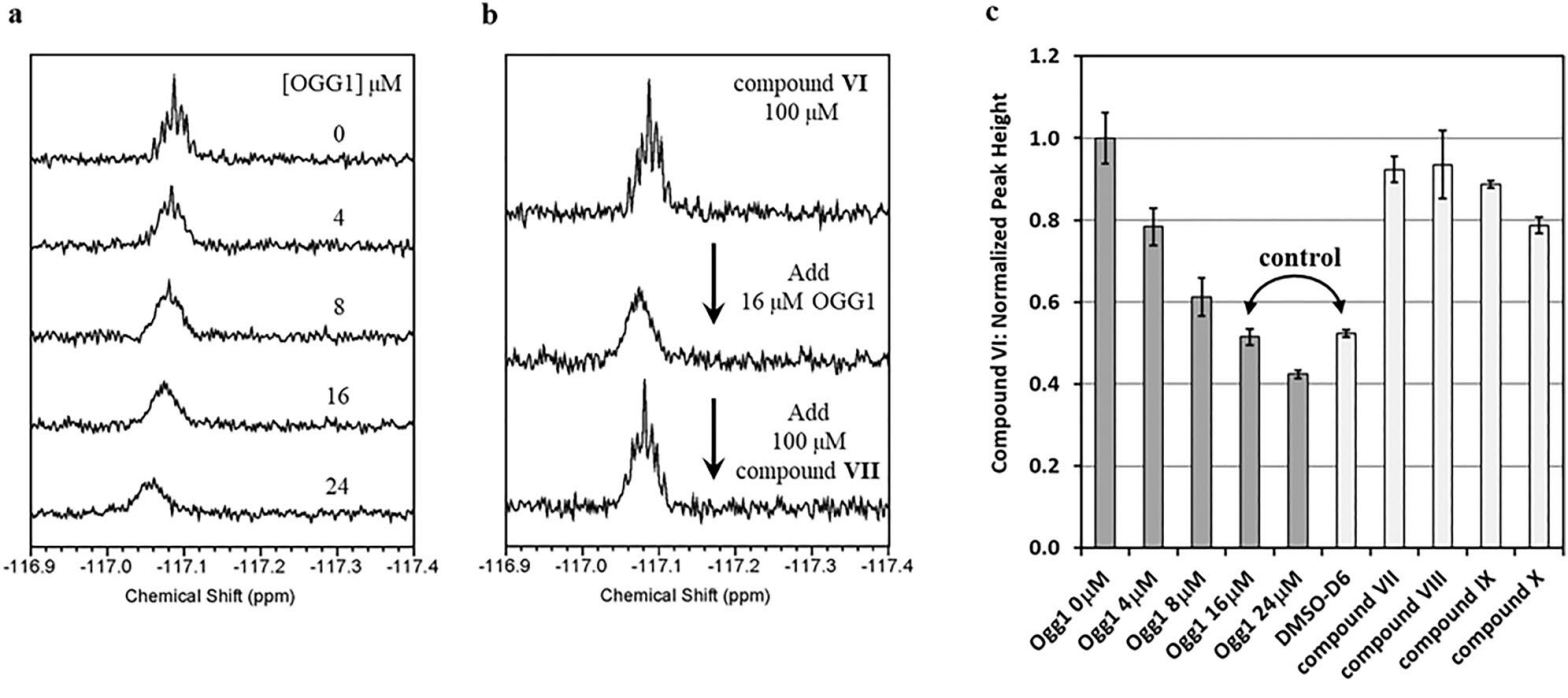
^19^F NMR Binding Experiments. (**a**) Non ^1^H-decoupled ^19^F NMR spectra of compound **VI** in the presence of different concentrations of OGG1. Gradual line broadening of the ^19^F NMR resonance as OGG1 concentration is increased indicates an interaction with OGG1. (**b**) Non ^1^H-decoupled ^19^F NMR spectra of 100 μM compound **VI** in the absence of OGG1 (top), presence of 16 μM OGG1 (middle) and presence of 16 μM OGG1 and 100 μM compound **VII** (bottom). The line narrowing effect observed on the bottom spectrum of Compound **VI** when compared to the middle spectrum is indicative of the two compounds competing for the same binding site. (**c**) Bar graph of the average peak heights extracted from the ^19^F NMR spectra (N = 2) of compound **VI** obtained with various OGG1 concentrations shown in panel **c** (dark grey on the left) and in the presence of 16 μM OGG1 plus 100 μM of a displacement compound, **VII**-**X** (light grey on the right). For the displacement experiments, the peak height of compound **VI** can be observed to increase, reflecting a line narrowing of its NMR resonance.

Compound **VI,** which contains a fluorine atom, was used in a ^19^F NMR analysis to detect compound binding to the free enzyme. Figure 4a shows that in the presence of OGG1 and a concentration range close to and above the *EC*_50_ for Compound **VI** (up to 24 μM), the ^19^F NMR resonance was broadened while also being shifted up field. This finding demonstrates that this compound binds to the OGG1 protein in the absence of a DNA substrate. Figure 4b shows a displacement experiment with compound **VII** in the presence of compound **VI** and OGG1. The line narrowing observed on the ^19^F NMR resonance of compound **VI** suggests that compound **VII** competes for the same binding site as compound **VI**. Similar results were obtained with compounds **VIII-X** as shown in Fig. 4c which illustrates the line narrowing effect through the peak height of the ^19^F NMR resonance of compound **VI**. These results further support an allosteric activation mechanism.

### Molecular modeling studies identify three potential sites for small molecule allosteric binders

**Figure 5:**
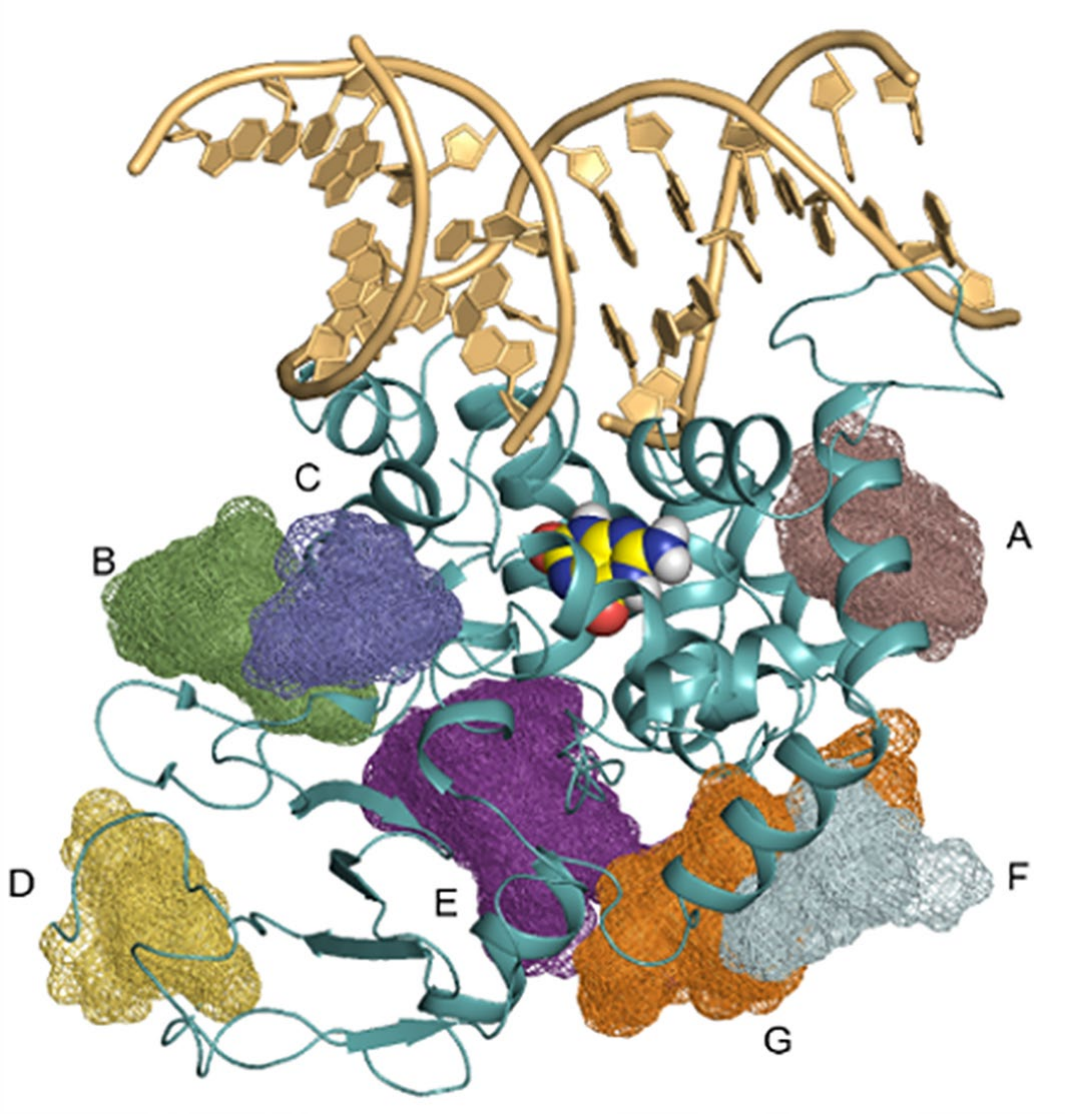
Proposed sites for small molecule OGG1 activator binding. OGG1 (blue ribbon) shown with 8-oxo-dG (yellow spheres) and DNA oligonucleotide (gold) from the cross-liked complex (PDB ID 1HU0). Hot spots (**A**–**G**) that were identified using mixed probe molecular dynamics were explored via docking experiments. Three hot spots, (B), (E), and (G) could accommodate > 9 small molecule activators.

Mixed probe molecular dynamics studies identified multiple sites which could accommodate small molecule ligands with appreciable affinities (< 10 μM). Docking studies were performed against seven of the largest sites (A-G in Fig. 5). After removal of highly strained poses (those ≥ 10 kcal/mol above the lowest energy conformation) only sites B, E, and G can accommodate at least nine of the ligands. All other sites can only accommodate five or fewer ligands.

### Cell-based studies demonstrate efficacy of OGG1 activators

Previously published work using compounds A, B, C, D and F (referred to herein as compounds **XI, XII, XIII, XIV** and **VII**, respectively, see Supplementary Table S3 online) showed that cells under oxidative challenge, i.e., paraquat (PQ), were protected from accumulation of 8-oxo-dG and associated PQ-induced cytotoxicity^27^. PQ, which at one time was a widely used agricultural herbicide, stimulated steady-state levels of 8-oxo-dG largely within the mitochondrial compartment. PQ is a redox cycler that associates with Complex I of the mitochondrial respiratory chain to produce the superoxide radical, which can then be transformed into hydrogen peroxide via superoxide dismutase^30^.

In the present studies, we examined the time- and concentration-dependent effects of PQ on ROS production. Supplementary Figure S2 (online) shows that ROS production was biphasic, with maximal levels obtained at 3 mM and 0.6 mM PQ at 24 and 48 h post-exposure, respectively, in A549 cells incubated with CM-H2DCFDA. Mitochondrial ROS production was also detected using the mitochondrial superoxide sensor, MitoSOX™ (Supplementary Fig. S2). PQ exposure to cells (0.03–3 mM) resulted in a concentration-dependent rise of mitochondrial ROS with maximal signal intensity (1.75-fold) at 3 mM which yielded an EC_**50**_ value of 0.28 mM for PQ after 24 h of exposure. Measurements of total glutathione content in response to PQ also showed a time- and concentration-dependent decrease of this major cellular antioxidant. After 24 h of PQ (1 mM) exposure, total glutathione decreased from 21.59 ± 0.44 mM to 17.25 ± 1.14 mM or by 20.1% but after 48 h, it declined to 3.9 ± 0.07 mM or by 87.5%. Total cellular glutathione loss indicated the severity of the ROS production throughout the cells even though it seemingly emanated from within the mitochondrial compartment. The ROS generated in the mitochondria also impacted nuclear DNA by inducing nuclear double-stranded breaks that were detected by γ-H2-AX staining. These breaks were observed only at 48 h beginning at 0.1 mM PQ (Supplementary Fig. S2) and co-existed with the concentration-dependent translocation of NF-κB (Supplementary Fig. S2), consistent with its activation. PQ activates NF-κB in tissues like brain^31^ and lung^32^ via a direct interaction with TLR4/9 receptors. Recent data indicates that fragmented, expulsed mtDNA arising from oxidative damage promotes pro-inflammatory events such as NF-κB translocation^33^.

**Figure 6:**
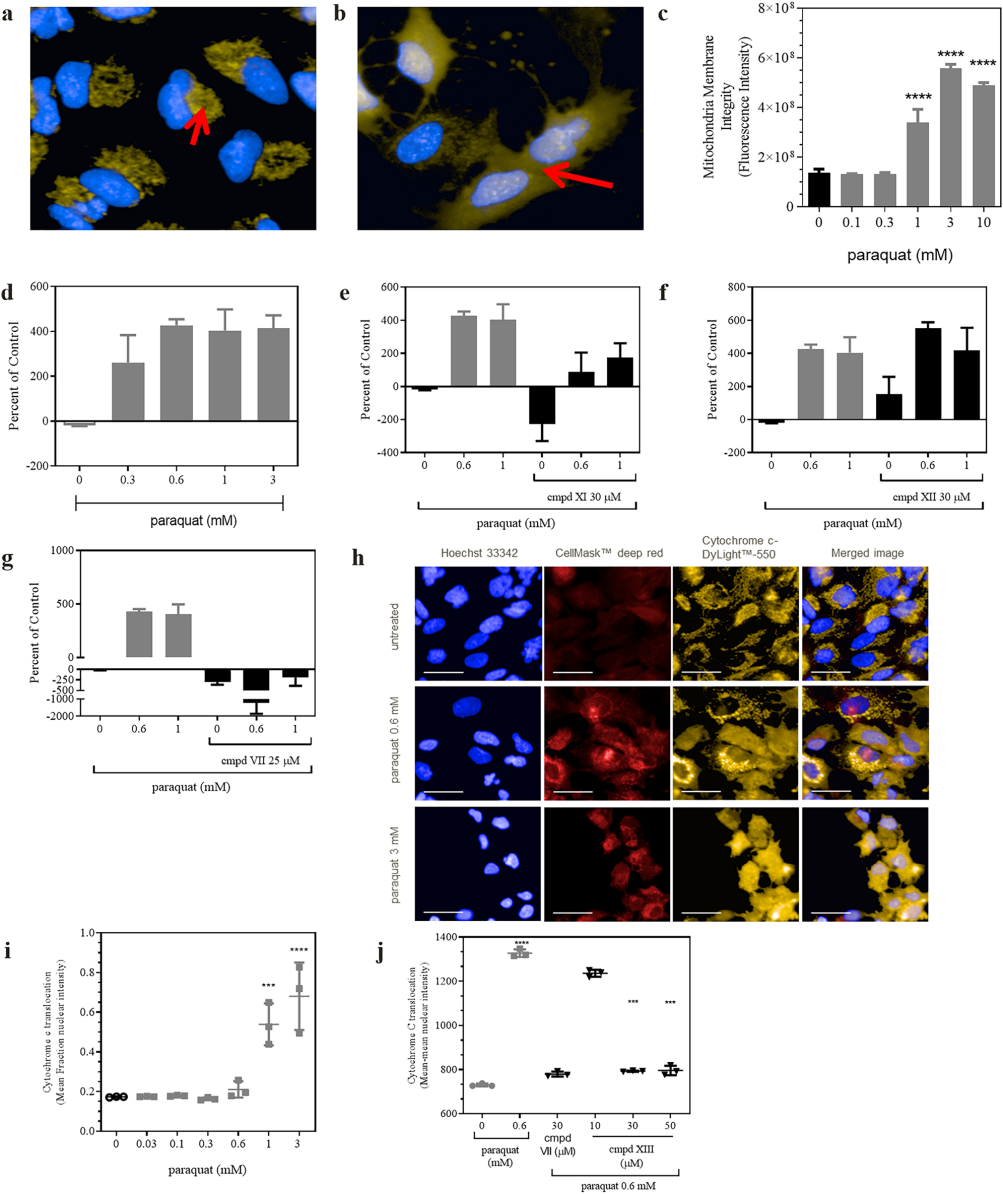
Effects of paraquat on mitochondrial membrane integrity, mtDNA damage and cytochrome *c* translocation. In (**a**) baseline values of mitochondrial integrity are noted by the punctate orange colors whereas in (**b**), paraquat exposure for 24 h elicits a diffuse characteristic in A549 cells. MitoTracker™ orange dye (overlaid orange) was used to demarcate mitochondria (red arrows) and Hoechst 33342 (blue) to denote the nucleus. (**c**) provides graphical representation of the mean values obtained from the imaging data from 3 independent experiments. Asterisks represent *p* < 0.0001 by 1-way ANOVA with Dunnet’s post hoc analyses. In (**d**), paraquat concentration-dependently damaged mtDNA as measured using two mitochondrial primers described in Methods. Values are depicted as percent of baseline control measurements. For (**e**–**g**), representative examples obtained from 3 independent experiments show the impact of compounds **XI**, **XII** and **VII**, respectively, on preventing mtDNA damage. For (**h**–**j**), changes in cytochrome *c* translocation are depicted in representative images using anti-cytochrome *c*-DyLight™-550 (yellow), CellMask™ Deep Red (red) and Hoechst 33342. In this case, OGG1 activators were applied 2 h prior to paraquat rather than 4 h in the other experiments. For (**i**) statistical analyses (ANOVA with Dunnett’s post-test), asterisks represent *p* < 0.0001 and for (**j**) PQ value was *p* < 0.0001 vs control while 30 and 50 µM of the test compound resulted in *p* = 0.0002 and 0.0003, respectively.

Although it can’t be ruled out that PQ stimulated ROS in areas other than the mitochondria, the physical tethering of the mtDNA to the inner mitochondrial membrane, which houses Complex I, renders both the mitochondrial membranes and the mtDNA a likely ROS target. The consequences of excessive ROS production on mitochondrial membrane integrity are depicted in Fig. 6. Using machine-learning software (PhenoLOGIC, PerkinElmer) and image acquisition of fluorescence arising from emission of MitoTracker orange dye overlaid within the cells, the normal punctate appearance of functional mitochondria was differentiated from the diffuse pattern exhibited by dysfunctional ones (Fig. 6c). The graphic representation shows that PQ evoked a concentration-dependent effect on mitochondrial membrane integrity with the maximum effect obtained at 3 mM. Using PCR and two mitochondrial primers to evaluate mtDNA damage^34^, PQ (0.3–3 mM) provoked a concentration-dependent rise in this important parameter (Fig. 6d). Maximal mtDNA damage was obtained at 0.6 mM PQ. From our previous work showing that the OGG1 activators prevented 8-oxo-dG accumulation^27^, we expected that the OGG1 activators would attenuate mtDNA damage. Figure 6e–g shows that the PQ-induced injury was markedly reduced by compounds **XI** and **VII** at both concentrations of PQ but not by compound **XIII**. The changes in mitochondrial membrane integrity and mtDNA damage were also associated with the concentration-dependent release of cytochrome *c* (Fig. 6h,i). Panel j shows that compounds **XIII** (30 and 50 μM) and **VII** (30 μM) essentially blocked this PQ (0.6 mM)-induced change. The discrepancy between the efficacy of compound **XIII** noted in the latter experiment and the lack of efficacy with mtDNA damage is not readily apparent at this time. Compound **XIII** was effective in preventing accumulation of 8-oxo-dG under similar conditions^27^.

In separate experiments, normal MEFs were challenged with 400 µM H_2_O_2_ to lower mtDNA content (Supplementary Fig. S3 online). At baseline, application of compound **VII** to MEFs elevated mtDNA levels above control values (Supplementary Fig. S3). After subjecting the cells to H_2_O_2_, mtDNA levels at 15 min of recovery were depleted to the same extent in control and compound **VII**-treated cells, however, compound **VII** samples achieved recovery faster (Supplementary Fig. S3). These data and those described above suggest that OGG1 activation by small molecules improves mtDNA maintenance under oxidative stress conditions.

Mitochondrial bioenergetics in A549 cells in response to PQ produced time- and concentration-dependent changes (Table 1). At 24 h post-incubation with the A549 cells, ATP levels fell from 6.29 ± 0.83 μmol/gm protein obtained in the absence of PQ to 3.13 ± 0.14, 2.49 ± 0.67, and 1.99 ± 0.45 μmol/gm protein in its presence. By 48 h of incubation, ATP levels further deteriorated, for example, at 3 mM PQ, the values decreased from 5.52 ± 0.81 to 0.73 ± 0.12 μmol/gm protein. By contrast, ADP rose from 17.18 ± 1.94 to 68.69 ± 1.47 μmol/gm protein for the same time period and concentration of PQ. In separate but similar experiments using lysates prepared from cells pre-incubated with compounds **XI**, **XIII**, or **VII**, for 4 h prior to PQ exposure, measurements of ATP and ADP (obtained from independent wells after 48 h of PQ exposure) indicated that the fall in ATP and the rise in ADP provoked by PQ was partially attenuated. The levels of both energy metabolites were increased across the PQ concentration range in the presence of the activators. For example, at 3 mM PQ, the ATP concentration was 0.63 ± 0.05 μmol/gm protein with PQ alone but was 1.31 ± 0.11 μmol/gm protein for PQ plus compound **XI**. Similar findings were obtained for compounds **XIII** and **VII**, respectively, i.e., 0.96 ± 0.10 vs 1.37 ± 0.30 and 0.61 ± 0.09 vs 1.73 ± 0.08 μmol/gm protein. These differences at this PQ concentration represented increases of 108%, 43% and 184%, respectively, which were marked improvements in the amount of ATP. The differences in [ADP] between PQ (3 mM) alone and PQ plus activators were only 9, 13 and 54%, respectively. By diminishing the impact of PQ on mitochondrial bioenergetics, the OGG1 activators maintain cell health, consistent with our earlier results^27^.

**Table 1:** Effects of OGG1 activation on paraquat-induced changes in energy metabolites.

Paraquat [mM]						Compound **XI** 30 µM				
	0	0.3	0.6	1	3	0	0.3	0.6	1	3
ATP Average (µmols/g protein)	3.18	1.89	1.19	1.02	0.63	3.93	2.90	2.89	2.67	1.31
ATP SEM	1.02	0.06	0.08	0.06	0.05	0.03	0.23	0.24	0.05	0.11
t-test (*p*-value; n = 3)							**0.0129**	**0.0026**	**0.00004**	**0.0042**
ADP Average (µmols/g protein)	11.55	19.72	39.71	46.80	53.45	11.08	29.20	37.95	35.40	58.12
ADP SEM	1.17	2.17	3.97	1.48	4.83	0.66	1.42	2.96	0.87	1.56
t-test (*p*-value; n = 3)							**0.0217**	**0.7402**	**0.0027**	**0.4102**
Paraquat [mM]						Compound **XIII** 30 µM				
	0	0.3	0.6	1	3	0	0.3	0.6	1	3
ATP Average (µmols/g protein)	4.60	2.56	1.43	1.47	0.96	2.86	2.59	2.22	2.20	1.37
ATP SEM	0.68	0.10	0.13	0.15	0.10	0.09	0.05	0.06	0.14	0.30
t-test (*p*-value; n = 3)							**0.8421**	**0.0048**	**0.0245**	**0.2612**
ADP Average (µmols/g protein)	7.49	1.49	13.54	22.34	40.48	7.80	13.61	31.29	2.20	45.92
ADP SEM	0.40	0.15	0.76	0.90	0.91	0.64	0.52	2.40	0.42	3.36
t-test (*p*-value; n = 3)							**0.00002**	**0.0021**	**0.0006**	**0.1931**
Paraquat [mM]						Compound **VII** 25 µM				
	0	0.3	0.6	1	3	0	0.3	0.6	1	3
ATP Average (µmols/g protein)	4.94	2.83	1.67	0.92	0.61	4.66	3.24	3.22	2.88	1.73
ATP SEM	0.28	0.07	0.11	0.06	0.09	0.21	0.03	0.17	0.28	0.08
t-test (*p*-value; n = 3)							**0.0065**	**0.0015**	**0.0023**	**0.0007**
ADP Average (µmols/g protein)	12.64	7.86	19.20	27.62	41.33	13.02	25.70	36.23	42.04	63.47
ADP SEM	0.76	0.50	0.66	1.66	3.06	0.35	1.18	1.47	0.76	1.86
t-test (*p*-value; n = 3)							**0.0002**	**0.0005**	**0.0014**	**0.0035**

A549 cells were cultured as described in Methods. Compounds were added to the cells 4 h prior to paraquat and were incubated for an additional 48 h prior to lysis for measurement of metabolites. Data represents mean ± S.E.M (N = 3 experiments with triplicate measurements). Statistical comparisons were made using an unpaired, two-tailed t-test between cells plus or minus paraquat. The *p-*values (significance) are in bold.

**Figure 7:**
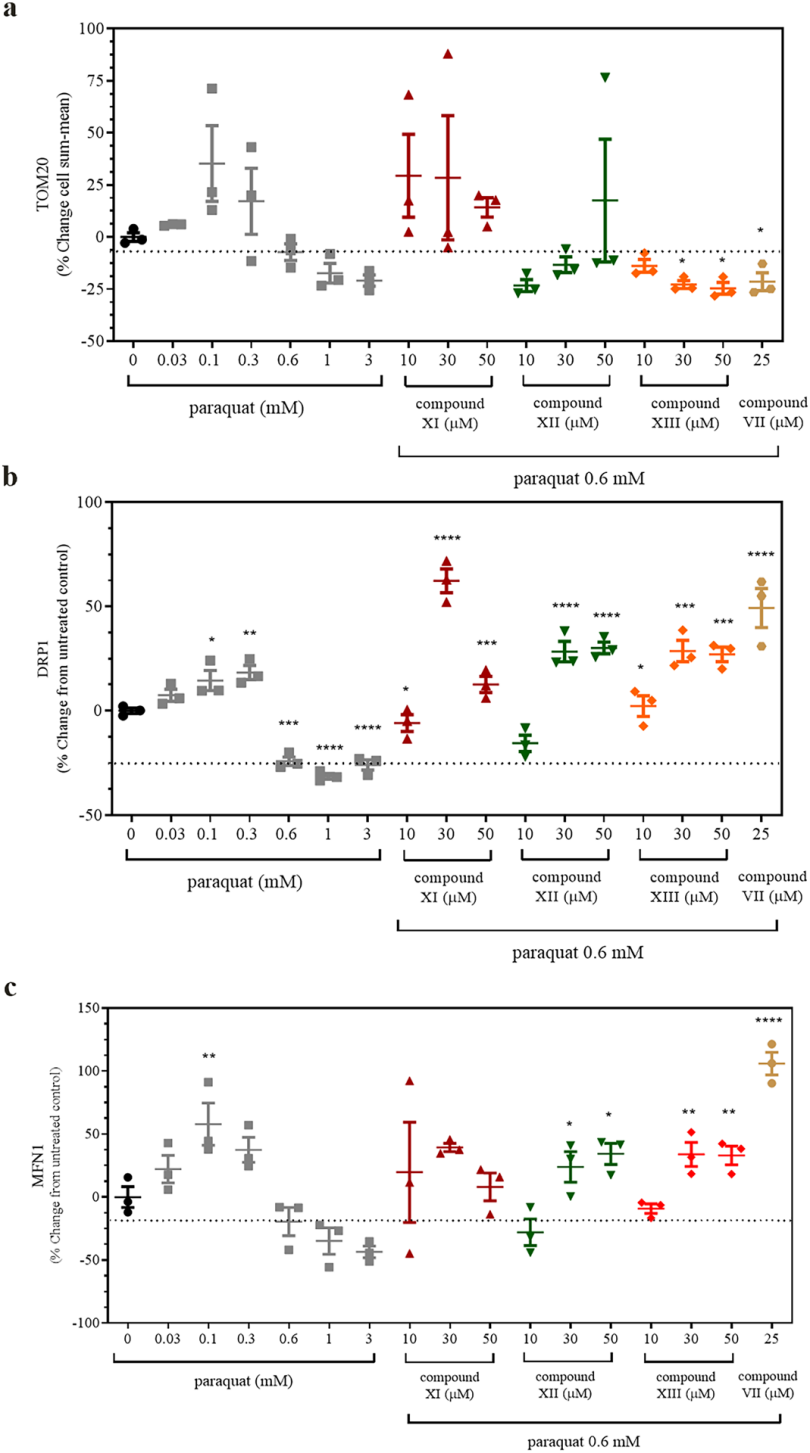
Paraquat-induced changes on mitochondrial dynamics. A549 cells were exposed to paraquat for 48 h and/or pre-treated with OGG1 activators for 4 h. Images were obtained and from these images graphical representations are displayed for (**a**) TOM20, (**b**) DRP1 and (**c**) MFN1. The dotted lines refer to the level of change induced by 0.6 mM paraquat. Values represent the average of two independent experiments in triplicate. Statistical significance was conducted and denoted as described in Fig. 6. For compound **VII** in (**a**), *p* = 0.042. In (**b**) *p* values from left to right side of the panel denoted by the asterisks equal 0.0138, 0.0023, 0.0002, < 0.0001, < 0.0001, 0.0242, 0.0002, 0.0003 and < 0.0001, respectively. For (**c**) the asterisks from left to right of the panel represent *p*-values of; 0.0099, 0.0498, 0.0183, 0.0045, 0.005 and < 0.0001, respectively.

Acute, low levels of PQ have been reported to adversely affect mitochondrial dynamics including excessive levels of fission and mitophagy with loss of TOM20 and other mitochondrial markers such as cytochrome oxidase IV in cultured neuronal cells^35^. The translocase of the outer mitochondrial membrane (TOM, TOM20 is a major subunit of TOM) is responsible for the import of nuclear-encoded proteins into the mitochondria. In the present study, PQ (0.03–3 mM) resulted in varied changes in TOM20 immunofluorescence (overlaid with images of MitoTracker™ orange and Hoechst 33342) in A549 cells. At low PQ concentrations (0.03–0.3 mM) TOM20 immunofluorescence increased to nearly 25% above baseline after 48 h of incubation of PQ at 0.3 mM PQ but then abruptly dropped below baseline values at 0.6–3 mM PQ (Fig. 7a). Preliminary studies indicated that the levels of dynamin related protein 1 (DRP1) in A549 cells was time-dependent over 18, 24, 30 and 48 h of incubation with PQ. At 48 h, DRP1 values fell below baseline beginning at 0.6 mM PQ (Supplementary Fig. S4 online). Similar to DRP1, A549 cells exposed to PQ revealed a time-dependent change over 24, 30 and 48 h with mitofusin 1 (MFN1) levels rising at the earlier timepoints and falling at 48 h, particularly at the higher PQ concentrations (Supplementary Fig. S4).

Application of the OGG1 activators; **XI**, **XII**, **XIII**, and **VII** produced variable results on TOM20 fluorescence. Relative to the mean value of 0.6 mM PQ alone, PQ plus compound **XI** (10, 30 or 50 μM) resulted in markedly higher values. PQ plus compound **XII** showed a concentration-dependent rise in TOM20 immunofluorescence whereas compound **XIII** (10, 30 or 50 μM) and **VII** (25 μM) had few effects. The impact of PQ plus or minus the OGG1 activators on DRP1 content using quantitative imaging is shown in Fig. 7b. When the experiment was repeated using compounds **XI**, **XII**, **XIII** and **VII**, applied 4 h prior to PQ with 0.6 mM PQ, Fig. 7b shows that DRP1 content declined by about 24 ± 0.02% at 48 h compared to control levels. By contrast, DRP1 content was increased, essentially at all concentrations of the OGG1 activators which suggested that OGG1 activation may promote mitochondrial fission. To determine whether mitochondria were also undergoing re-organization and fusion, we evaluated the marker MFN1. Increased expression of *MFN1* and *MFN2* together with enhanced mitochondrial elongation have been identified in animals over-expressing mitochondrial OGG1^18^. Figure 7c demonstrates that compounds **XI**, **XII**, **XIII**, and **VII** (4 h pre-PQ) also elevated MFN1 protein content after 48 h of PQ (0.6 mM) exposure. The highest levels of MFN1 protein were obtained with compound **VII** (25 μM), about 106% and 49% above basal and PQ levels, respectively. These data above are indicative of OGG1 mediated mitochondrial re-organization.

**Figure 8:**
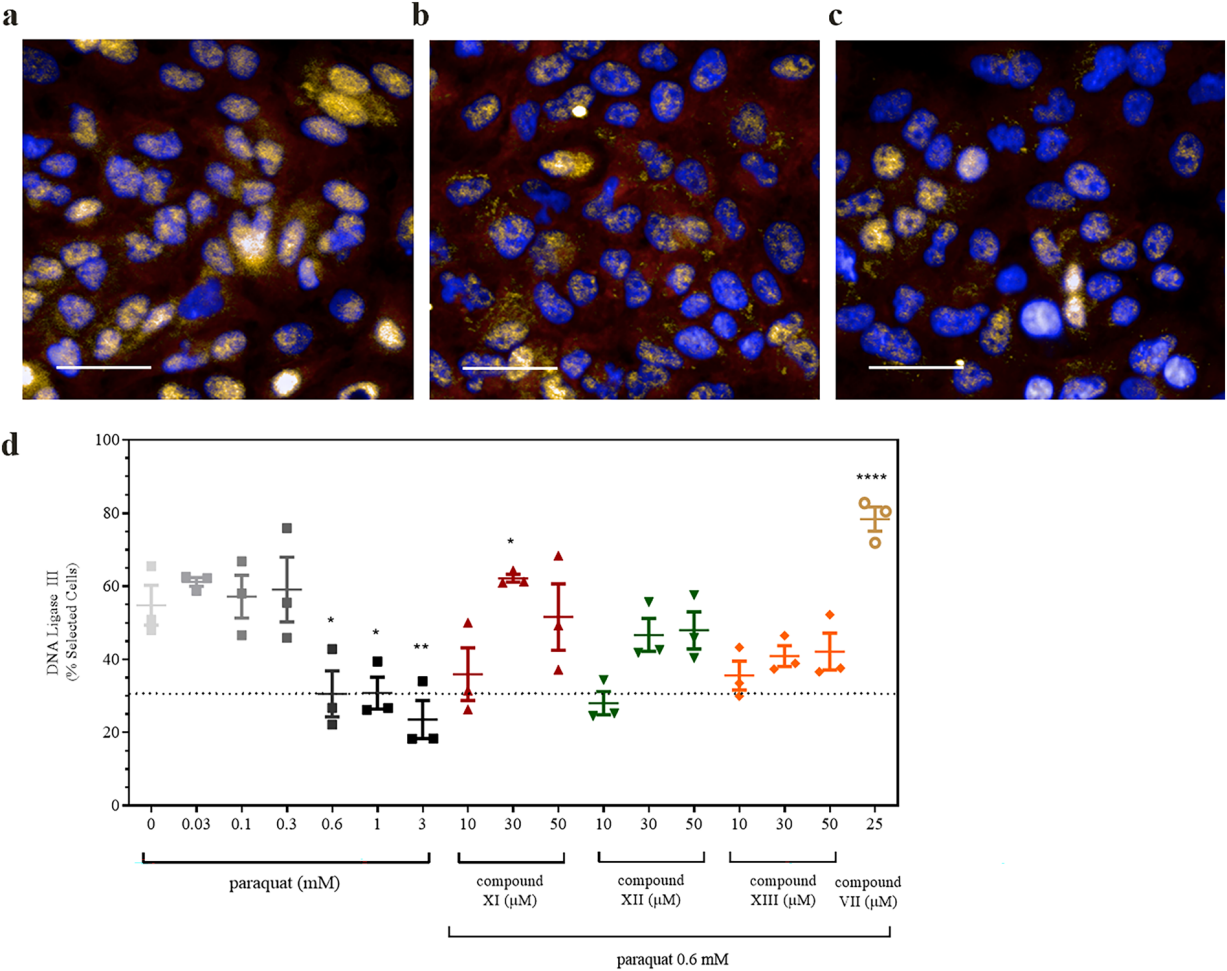
Effects of paraquat on DNA Ligase III in the presence and absence of OGG1 activators. Panels (**a**–**c**) represent images obtained in the absence and presence of 0.6 and 1.0 mM paraquat for 48 h, respectively. Sample images depict changes in DNA ligase III content (overlaid orange) with overlaid Cell Mask™ Deep Red (red) and Hoechst 33342 (blue). The line = 40 microns. In (**d**), graphical representation of the changes resulting in DNA ligase III content after exposure to 0.6 mM paraquat with the dotted line used to depict the changes brought about by the presence of the OGG1 activators (4 h pre-treatment). Similar data were obtained with 24 h of paraquat treatment (data not shown). Values are representative of two independent experiments performed in triplicate. In (**d**) *p*-values denoted by the asterisks from left to right of the panel equal 0.0427, 0.0448, 0.0083, 0.0239 and < 0.0001, respectively.

Excessive OGG1 activity could lead to the formation of apyrimic/apyrimidinic (AP) sites in the absence of sufficient participation by AP endonuclease 1 (APE1) and other downstream members of the BER pathway. Previous data using adenovirus to overexpress mitochondrial OGG1 in H9C2 cells exposed to oxidative stress showed that mRNA expression of BER members APE1 and DNA polymerase-γ was significantly upregulated by about twofold in the transduced cells compared to control ones^20^. In the present study, the protein content of DNA ligase III (LIG3), which may be a rate-limiting component of the BER pathway^36^, was measured. Figure 8 shows that LIG3 levels declined by about 50% in response to 48 h of PQ treatment (at either 0.6 or 1 mM). By contrast, LIG3 protein was significantly increased in the presence of each of the OGG1 activators. At 0.6 mM PQ, compound **VII** resulted in values that exceeded those values in its absence. In a separate experiment, 0.6 mM PQ increased OGG1 content by 88.3% above baseline (N = 1 in triplicate, *p* < 0.0001). In the presence of 0.6 mM PQ, two different chemotypes, compounds **VII** (30 µM) and **XI** (50 µM) increased OGG1 content by 142.3% (*p* = 0.0056) and 227.4% (*p* = 0.002), respectively, above that achieved with PQ alone. The previous findings described above together with those found in the present investigation suggest that enhanced OGG1 activity by an as yet unknown mechanism enhances the protein content of members of the BER pathway for mtDNA repair.

## Discussion

DNA damage arising from ROS, which are a natural by-product of mitochondrial respiration, has long been considered to be a contributing factor to aging and its accompanying diseases. The magnitude of ROS exposure in human cells each day necessitates robust repair systems particularly within the mitochondria. As such, BER and specifically OGG1, have received considerable investigative attention. The present work expands on mechanisms of enhanced OGG1 catalysis for removal of 8-oxo-dG from DNA. Using small molecule OGG1 activators, we also demonstrate potential advantages for mitochondrial function during oxidative assault.

Product-assisted catalysis was first proposed to describe enhancement of OGG1 catalysis^24^. The evidence for this unique mechanism (Fig. 1b i) was supported by the co-crystal structure of OGG1 with an oxidative lesion-containing DNA substrate. In this co-crystal structure, the cleaved nucleobase, 8-oxoGua, was sequestered at the product recognition pocket with the N9-atom oriented in a seemingly catalytically competent position. Although such a structural orientation would be consistent with product-assisted catalysis, it is not a proof per se of this mechanism. This co-crystal structure is likely a snapshot in time when 8-oxoGua exits the catalytic pocket after cleavage from the lesion-containing DNA substrate, as opposed to 8-oxoGua serving as a catalytic base to prompt subsequent β-elimination by the AP lyase reaction. The present studies demonstrate that activation of OGG1 can be obtained by 9-deazaGua. This product analog lacks an N9-atom that is absolutely required for product-assisted catalysis. Together with the observed random kinetic mechanism, our data suggest that product-assisted catalysis is not a likely mechanism of OGG1 activation in agreement with other investigators^25^. Moreover, the present data are fully consistent with a mechanism driven by allosteric activation (Fig. 1b ii). Finding structurally diverse small molecule activators, distinct from product analogs, through random screening and demonstrating binding of a small molecule activator (compound **VI**) to free enzyme, further supports an allosteric mechanism of OGG1 action.

Feedback regulation, either positive or negative, is an efficient mechanism for cells to adapt to fluctuations in dynamic biochemical pathways in response to an ever-changing cellular environment^37^. Positive feedback regulation of OGG1 by 8-oxoGua could be beneficial during oxidative stress. It has been argued that OGG1, although bifunctional, is mainly responsible for catalyzing the glycosylase reaction in the intracellular context that produces an abasic DNA product under normal physiological conditions^38^. Allosteric activation of OGG1 AP-lyase activity mediated by the product of the OGG1 glycosylase reaction and its analogs, as shown herein, may provide a positive feedback regulation mechanism to enhance BER in cells undergoing repetitive oxidative stress. When 8-oxo-dG accumulates, it may allosterically bind to OGG1 following excision and enhance AP-lyase functionality to accelerate BER. In turn, cells can accommodate and, over time, adapt to a more hostile environment. The content of OGG1 in the nucleus, perhaps at the cost of mitochondrial dysfunction, increased in cultured cortical neurons following short periods (4 h) of oxidative stress^39^. Therefore, the notion of a product-mediated allosteric activation of OGG1 offers a mechanistic explanation of the OGG1 AP-lyase catalysis but also provides a plausible mechanism by which cells respond to oxidative stress.

Allosteric activation of enzymes derives from either a reduction in *K*_m_, an increase in *V*_max,_ or a combination of the two as the result of activator binding to an allosteric binding site on the enzyme. The α value of ~ 1 determined from the initial rate analysis of OGG1 in the current study indicates that the allosteric activation of OGG1 by 8-oxoGua analogs (Fig. 3) is mainly due to an increase of *V*_max_. The maximum velocity for the OGG1 AP-lyase reaction involves dynamic processes that precede and include the chemical step (β-elimination), the chemical step itself and product dissociation^40^. The increase in *V*_max_ of OGG1 by 8-bromoGua arises from acceleration of the chemical step^24,25,40^. An activation level of more than tenfold by 8-bromoGua has been reported previously^24^ which is consistent with the β-value of 36 (Fig. 3) obtained in the current investigation.

Allosteric activation of OGG1 requires an enzyme-derived catalytic base for C2′-proton abstraction in the AP lyase reaction (Fig. 1b ii). A plausible candidate is Asp268. According to the structure of OGG1 co-crystalized with an 8-oxo-dG lesion-containing DNA substrate, there is only one residue within the active site that is capable of base catalysis, i.e., Asp268^24^. In this co-crystal structure, Asp268 is separated from C2′ by 4.9 Å, a distance that is too long for Asp268 to abstract a C2′-proton efficiently. However, it is conceivable that this distance could be shortened through a conformational change induced upon binding of an activator at an allosteric site. Norman and coworkers^41^ reported that changing Asp268 to Asn268 by site-directed mutagenesis did not significantly perturb the basal activity of strand cleavage. Activation of strand cleavage in the presence of 8-aminoGua, which was observed for the wild-type enzyme, was markedly compromised in the Asp268Asn protein^41^. This result suggests that Asp268 functions as the catalytic base for the AP lyase reaction. In the absence of activator, Asp268 is located so far from C2′ that it cannot be efficiently engaged in base catalysis. However, Asp268 could move closer to C2′ upon activator binding at an allosteric binding site. As such, Asp268 becomes fully competent for abstracting a C2′-proton. Although the evidence for the allosteric activation of OGG1 by product analogs and small molecule activators is compelling, the exact location of the allosteric binding pocket remains to be confirmed by co-crystallographic analysis of ligand binding.

In general, enzyme activators are more difficult than inhibitors to identify through high throughput screening. Small molecule activators that act as catalysts, i.e., organo-catalysts, are perhaps even more challenging to discover. They must bind to the active site, possess the unique catalytic group and adopt a precise orientation for catalysis. The current evidence argues against product-assisted catalysis and supports allosteric activation using a diverse set of small molecule activators identified through random screening. This validates targeting OGG1 with small molecule activators as potential drug candidates. Our mixed probe molecular dynamics studies suggest that several likely “hot spots” exist for allosteric OGG1 ligand binding. As shown in Fig. 5, sites B, E, and G on the OGG1 protein provide potential starting opportunities for future investigation. The importance of the molecular docking experiments against these hot-spot sites is threefold: (1) the three identified sites (B, E, and G) which accommodate the diverse ligands in this study sit outside of the catalytic pocket, thereby defining the allosteric interaction sites between small molecules and OGG1 for organo-catalysis, (2) they suggest a path forward for additional experiments to resolve the interactions of the identified chemophores using physiological concentrations of ligand during soaking with the OGG1 crystal structure, and (3) limits the search space to three sites instead of ten for additional first-principles (e.g., free energy perturbations) and machine learning approaches to optimize ligands in sites B, E, and G.

Some of the small molecule OGG1 activators described in the current work were previously shown to protect cells from accumulating PQ-induced 8-oxo-dG in mtDNA^27^. These earlier findings indicated that mitochondrial 8-oxo-dG formation was associated with loss of mitochondrial membrane potential (MMP), which is vitally important for energy production. The OGG1 activators attenuated this outcome and as shown here, preserved ATP levels. Moreover, the PQ-stimulated ROS occurred concomitantly with loss of mitochondrial membrane integrity and mtDNA damage in human A549 cells. Damage to mtDNA was diminished by two of the OGG1 activators. The direct application of H_2_O_2_ to a different cell type, i.e., MEF cells, decreased mtDNA copy number (CN) and its recovery was accelerated by the presence of the OGG1 activator, compound **VII**. DNA strand breaks, particularly mtDNA, lead to fragmentation and expulsion to elicit inflammation through several pathways, such as NF-κB translocation^42^. PQ also induced NF-κB translocation. The pretreatment of A549 cells with the OGG1 activators also offset cytochrome *c* translocation which precedes apoptosis. Although the TOM20 results did not indicate enhancement of mitochondrial density, the greater DRP1 and MFN1 protein levels in the presence of the OGG1 activators suggest heightened mitochondrial re-organization to preserve mtDNA integrity. The increased LIG3 and OGG1 content in response to PQ and the OGG1 compounds likely promotes improved BER activity to assure efficient repair of the damaged nucleobase. Lastly, cellular efficacy of these diverse “tool” compounds may be influenced by molecular weight, size, charge, lipophilicity and chemical stability in saline media.

The most prominent mitochondrial function is energy production, which is intimately dependent upon MMP. Recent studies suggest that OGG1 influences MMP. In cultured cortical neurons isolated from mice lacking the genes, *Mth1* and *Ogg1*, 8-oxo-dG accumulated in mtDNA which was accompanied by a loss of MMP and mitochondrial function^43^. OGG1 siRNA rapidly decreased α-OGG1, which has a targeting sequence for mitochondrial import, coincident with progressive deterioration of MMP with menadione-induced mitochondrial superoxide production^44^. Respiratory capacity was, however, unaltered using OGG1 deficient (KO) mice^45^. Decreased mitochondrial function was unrelated to mass and due to fragmentation, suggesting that α-OGG1 is “essential to mitochondrial morphology and function after exogenous oxidative stress”^44^. Our previous investigation^27^ and the present one, are consistent with this perspective.

The accumulation of 8-oxo-dG lesions within DNA triggers serial events that are deleterious to mitochondrial function and cell viability. Menadione-induced oxidative stress in OGG1 KO cells provokes rapid cell death by two separate mechanisms, i.e., poly-ADP-ribose polymerase-dependent or calpain-mediated^46^. Single-stranded breaks in both genomes precedes cell death whereas breaks in mtDNA provoke its depletion. In turn, the loss of mtDNA CN lowers energy production to compromise cell viability^34^. By contrast, overexpression of mitochondrial OGG1 lowers mtDNA 8-oxo-dG levels with fewer mtDNA deletions and fragments with improved CN to support expression of mitochondrial encoded proteins, preservation of MMP and reduce apoptosis^20^. Sufficient CN is absolutely necessary for biogenesis of key oxidative phosphorylation proteins^47^. Depletion of CN impedes mtDNA replication^47^, while the persistence of abasic sites blocks replication^48^. Heteroplasmy and low levels of absolute CN may influence the onset of human age-related diseases^49^, whereas high levels of wild type mtDNA can offset deleterious pathogenic mtDNA mutations^50^ and support organ function^51^. Thus, the protection against PQ-induced mtDNA damage and the accelerated recovery of CN post-H_2_O_2_ exposure by our OGG1 activators may provide a therapeutic means to slow disease pathology.

The metabolic and functional ramifications of OGG1 deficiency (KO) or its overexpression, particularly within mitochondria, have been examined in several murine models of disease. As compared to wild type mice, OGG1 KO mice have 20-fold more 8-oxo-dG in their mtDNA^52^, suffer greater levels of ischemic cortical infarct^39^ and with aging display a Parkinson’s disease phenotype^53^. Moreover, the KO mice have greater high fat diet (HFD)-induced adiposity with metabolic syndrome features^54^ and reduced gene expression of Krebs’ cycle components. By contrast, transgenic mice that overexpress mitochondrial OGG1 have lower levels of 8-oxo-dG within white adipose tissue, improved whole body energy expenditure and attenuated adiposity^18^. Mitochondria of OGG1 transgenic mice are elongated with increased expression of mitochondrial encoded genes with reduced levels of NF-κB. The latter phenotype indicates that enhanced OGG1 activity within mitochondria improves their function when stressed.

Collectively, our previous^27^ and current findings demonstrate that OGG1 represents a promising drug target for discovering small molecule activators to treat oxidative stress-related diseases. Potentially contributing to these diseases are genetic and environmental factors that lower OGG1 efficacy. For example, activity by the loss of function OGG1 *Ser326Cys* variant, which is associated with Alzheimer’s Disease^55^ and all-cause mortality^56^, is improved by our small molecule OGG1 activators^27^. ROS-mediated oxidation of OGG1’s multiple cysteines or exposure to cadmium also lowers OGG1 catalysis^57,58^. Independent confirmation that small molecules can enhance OGG1 activity^59^, albeit by a separate mechanism than that described herein, i.e., via polynucleotide kinase phosphatase, strongly indicate that OGG1 is druggable. It is noteworthy, however, that some pro-inflammatory conditions may benefit from OGG1 inhibition^60,61^. The OGG1 inhibitor, TH5487, prevents TNF-α-induced OGG1 interactions with nuclear DNA to decrease NF-κB binding^62^. Oxidized mtDNA within mitochondria, if not readily repaired by OGG1, fragments and exits the organelle to initiate inflammation^63^. Nonetheless, allosteric activation by small molecules is favorable to drug discovery. The further optimization of these early, tool compounds toward selective, orally available, and potent small molecule OGG1 activators along with comparison to their inhibitory counterparts will promote greater understanding of their therapeutic potential.

## Materials and Methods

Unless otherwise stipulated, chemicals including 8-bromoGua, 8-aminoGua, and Benzonase® nuclease were purchased from Sigma-Aldrich (St Louis, MO), Roche (San Francisco, CA) or for 9-deazaguanine (9-deazaGua), Santa Cruz Biotechnology (Dallas, TX). The purity of 9-deazaGua was > 99.5% as determined by LCMS (Supplementary Fig. S5).

### Preparation of human recombinant OGG1

As described previously^27^, *Escherichia coli* cells over-expressing Flag-His-Tev-rhOGG1 (amino acids 1–345) were lysed in buffer A (50 mM Tris–HCl, pH7.5, containing 250 mM NaCl, 25 mM imidazole, and 0.1 mM TCEP) supplemented with Benzonase® nuclease and EDTA-free, complete, protease inhibitor cocktail tablets. The lysate was passed twice through an Avestin pressure drop homogenizer (12 kpsi). Cell debris was removed by centrifugation (30,000 g X 45 min) and the supernatant mixed with Ni–NTA agarose resins equilibrated with buffer A. The mixture was rotated (2 h), centrifuged (4,000 g X 5 min), resuspended in buffer A and packed into a XK26 column, which was then washed with buffer A until *A*_280_ reached baseline. The captured OGG1 protein was eluted with 0–50% buffer B (buffer A + 500 mM imidazole) gradient in buffer A. The eluted fractions were analyzed by SDS–PAGE. Fractions containing protein were pooled and concentrated using a 10-kDa molecular weight cut-off centrifugal concentrator. The tags of the OGG1 protein were removed by His6-Tev protease (enzyme: substrate ratio = 1:100) followed by passage through a Ni–NTA agarose column. Tag-free OGG1 was collected, concentrated, and injected into a Superdex 200 26/60 gel filtration column equilibrated with buffer A. The eluted protein was detected by a 4–12% gradient SDS–PAGE. Human OGG1 fractions were pooled, aliquoted and stored in buffer B plus 400 mM NaCl at − 80 °C. Both the full length (1–345 amino acids) and truncated (12–345) OGG1 proteins were prepared to 99% purity without DNA contamination measured by gel densiometry with chromatograms at an A280/A260 ratio. Both proteins were tested using a gel-based assay and the fluorescent kinetic assay described below, with comparable activity, however the truncated form was used in these studies. It has been reported that the OGG1 β-isoform in situ lacks glycosylase activity but protects mitochondrial function as an aconitase chaperone protein^64^.

### Fluorescent steady state kinetic assay

An assay was constructed based on published methods^28^ using an 8-oxodG-containing hairpin oligodeoxyribonucleotide (IDT, Coralville, IA) in water (100 μM) which was annealed (Peltier Thermal Cycler, BIO-RAD, Hercules, CA) by heating (90 °C X 5 min) and cooling (4 °C at 0.05 °C/sec) the sample. All reactions (total assay volume = 10 μl; the buffer contained 50 mM Tris–HCl, pH 7.5, 50 mM KCl, 1 mM EDTA, 1 mM DTT, 1 mM CHAPS and 0.1 mg/ml BSA) were run in Greiner 384 black low volume plates (Bio One, Monroe, NC). The annealed substrate plus or minus activator was mixed with enzyme and the evolving fluorescence of each well was measured in kinetic mode (λ_ex/em_: 485/522 nm and a dichroic at 515 nm, SpectraMax, Molecular Devices, San Jose, CA). Initial rates were calculated from the linear phase and the molar concentration of the reaction product from a FAM standard curve.

### Compound screening

Compounds (> 2 million from the GSK collection) were dispensed (0.06 μl/well in DMSO) in 1536 Greiner black low volume plates. The 8-oxodG containing hairpin DNA substrate in 3 μl assay buffer was added to each plate well followed by addition of 3 μl enzyme. The concentration of the reactants in the compound screening protocol were: OGG1 = 10 nM, substrate A = 100 nM, and compounds were added at 20 µM. The level of DNA cleavage was: < 5% for OGG1 alone and about 20% for OGG1 + Hits or 8-bromoGua. Compounds with activation > 20% relative to that obtained with 500 μM 8-bromoGua, which was defined as 100%, were classified as potential Hits. After incubation (22 °C X 40 min), fluorescence intensity was determined (ViewLux, Perkin Elmer, Waltham, MA). Concentration responsiveness was evaluated with these hits followed by a least squares analysis according to Eq. (1):

1 $$\nu = \frac{{E_{\max } - B}}{{1 + \left( {\frac{{EC_{50} }}{{\left[ {\text{A}} \right]}}} \right)^{n} }} + B$$

where *ν* = the initial rate in the presence of an activator, *E*_max_ = maximum activity, *EC*_50_ = concentration of activator at which the enzyme displays half-maximal activity, *B* = background, and n = the Hill coefficient. Several Hits were assessed for binding to OGG1 using surface plasmon resonance (Biacore T100, A100 and S51, Cytiva, Sweden). For representative values of compounds used in cell-based studies, see Supplementary table 3 legend. Each compound was measured for its binding characteristics to an immobilized target as well as non-specific interactions with decoy proteins, e.g., glutathione, and a blank sensor chip surface. Ogg1 specificity is addressed by subtracting the binding response from a reference cell (Neutravidin surface) from the surface containing the target protein (OGG1 plus Neutravadin surface).

### ^19^F-NMR of OGG1 binding by small molecule activators

Samples were prepared in 20 mM Tris–HCl, pH 7.5, containing 150 mM NaCl, 0.1 mM TCEP, 2% DMSO-D_6_ (99.9 atom %D) and 20% D_2_O (99.9 atom %D). Line broadening of a single fluorine-containing OGG1 activator (compound **VI**, 100 μM) upon binding to OGG1 (0–24 μM) was monitored at 564.17 MHz by ^19^F-NMR (Varian Unity INOVATM 600 system, Agilent Technologies, Santa Clara, CA). Line narrowing resulting from displacement of the bound fluorine-containing compound was also measured in the presence of a potential competitive displacement compound e.g., compound **VII** (100 μM). ^19^F-NMR spectra were recorded using a 5 mm fluorine probe, regulated at 20 °C and the following parameters: 128 K complex data points, sweep width of 139,860 Hz, acquisition time of 0.469 s per scan, 384 scans and a relaxation delay of 1.85 s. Prior to Fourier transformation, sensitivity and resolution were enhanced by applying an exponential line broadening of 1 Hz over the first 128 K data points and zero filled to 512 K data points. Spectra were analyzed using in-house data analysis software.

### Molecular modeling of OGG1 to identify potential small molecule binding sites

To identify potential small molecule binding sites, mixed probe molecular dynamics were carried out as previously described^65^ using the published OGG1 crystal structure^66^. The simulation was carried out with a box of 80% water and 20% probes (14% isopropanol with 2% each of acetamide, isopropylamine, and acetate plus one sodium counterion to neutralize charge)^24^. An equilibration phase of 1.5 ns was followed by a 40 ns simulation. The threshold for a site to be a potential binding “hot-spot” was set to < -1 kcal/mol. Modeling small molecule interactions with OGG1 was accomplished using GlideXP^67^ as implemented in the 2017–4.11 Schrodinger Suite with OPLS2005^68^ forcefield. OGG1 from the crystal structure^24^ was protonated (pH 7.4), whereas missing rotamers and contiguous gaps less than 5 amino acids were rebuilt using Prime^69^. Crystallographic waters greater than 5Å from the nearest sidechain were removed. For docking experiments, protein grids centered at each hot-spot were prepared (box length = 10Å—14Å, depending on pocket solvent exposure) and a rigid receptor was used. Ligands were protonated and tautomerized to pH 7 (± 2) with those having a probability > 5% kept as distinct entities for docking with up to 500 conformations/ligand. Each minimized docked pose was visually inspected. Those with nonsensical interactions or obviously strained geometries were removed. Docking-induced strain was calculated by assessing the distance between global and local minima (OPLS2005).

### Cell culture

Type II alveolar epithelial cells, A549 (ATCC; Manassas, VA), were grown to sub-confluence as described previously^27^ using complete Ham's F12 medium with 10% heat-inactivated fetal bovine serum (FBS), and GlutaMAX™ supplement (Life Technologies, Carlsbad, CA) in O_**2**_:CO_**2**_; 95:5% at 37 °C. For sub-culture, the cells were treated with Trypsin/EDTA (Lonza Inc., Walkersville, MD), counted (Trypan blue, Life Technologies, Carlsbad, CA), and seeded (100,000 cells/ml in 96-well plates). Oxidative stress was invoked by paraquat (N, N′-dimethyl- 4,4′-bipyridinium dichloride) at varying concentrations (0.01–3 mM) and incubation periods (refer to figure legends). PQ was dissolved in Dulbecco’s phosphate buffered saline and stored in aliquots (1 M, − 20 °C) until use when they were thawed and diluted in cell culture medium. Unless stated otherwise, OGG1 activators were incubated with cells for 4 h prior to treatment with PQ. All measures were performed in triplicate on three independent occasions unless stated otherwise. For specific details describing the immunofluorescence staining and quantification please refer to the Supplemental materials.

Mouse embryonic fibroblasts (MEFs) were grown in Dulbecco’s Modified Eagle Media (25 mM glucose with pyruvate; ThermoFisher, Waltham, MA) with 10% FBS at 5% CO_2_ at 37 °C. For sub-culture, cells were trypsinized (ThermoFisher, Waltham, MA), counted using a cytosmart cell counter (Corning, Corning, NY), and 100,000 cells were plated per well in 12-well plates. Cells were cultured for 24 h prior to a 4 h treatment with 30 µM compound (in DMSO) or vehicle. For hydrogen peroxide (H_2_0_2_) treatments, the stimulus concentration was determined by the absorbance at 240 nm (1.31 = 30 mM H_2_0_2_) and adjusted in calculations. Cells were washed with PBS and exposed to 400 µM H_2_0_2_ in DMEM without FBS for 15 min. At that time, H_2_0_2_ was neutralized by the addition of an equal volume of DMEM with 2X FBS, all media removed, cells washed with PBS, and warm DMEM with FBS added for the indicated recovery time. To collect cells, plates were washed with PBS, trypsinized and collected by centrifugation prior to flash freezing in liquid nitrogen. All samples were collected and kept at -80˚C until analysis for mtDNA determination. MEFs were kindly provided by Dr. Mark Gladwin, University of Pittsburgh Medical Center. All work regarding the collection of the MEFs were conducted in accordance with Institutional Policy on the Care, Welfare and Treatment of Laboratory Animals and were reviewed by the Institutional Animal Care and Use Committee using an ethical review process at the institution where the work was performed.

### Quantitative mtDNA and mtDNA damage determination

MEFs pellets were rapidly thawed and digested with SDS/proteinase K as previously described^70^. After freeze–thaw of resuspended DNA, total DNA was quantitated by AccuBlue® (Biotium, Freemont, CA) and assayed for relative nuclear DNA (nDNA) levels by quantitative PCR as described^71^. Samples were adjusted to be within + /- 1 Cq value and assayed in duplex for both mtDNA (mitochondrial NADH-dehydrogenase subunit 1; mt-ND1) and nDNA (HDAC1) against a pooled sample standard curve. Both nuclear and mitochondrial targets were plotted and validated to be in the linear range. Furthermore, ∆Cq values were plotted to assess the effect of dilution on relative mtDNA levels. With these validations in place, we determined the relative mtDNA levels (normalized to nDNA levels).

For mtDNA damage, A549 cells were lysed using the Cells-to-Ct kit (ThermoFisher, Waltham, MA). DNA was isolated, quantified and followed by long and short chain PCR reactions according to published methods^34,72^. Long-chain PCR was conducted using the 8.9 kilobase mtDNA fragment and primers (IDT, Research Triangle Park, NC) with accession numbers 14841 and 5999; and for short-chain PCR using the small mt fragment and accession numbers 14620 and 14841^34^. The PCR product was quantified using PicoGreen™ (Life Technologies, Carlsbad, CA). Fluorescence intensity of the long-chain was subtracted from that of the short-chain and expressed as a percent of the untreated control.

### Measurement of energy metabolites

Adenosine triphosphate (ATP) and adenosine diphosphate (ADP) levels in A549 cells cultured in 6-well plates were measured following treatment with or without PQ (0–3 mM, plus or minus OGG1 activators) using commercially available kits (BioVision, Milpitas, CA and Abcam, Cambridge, MA, respectively). Samples were collected from separate plates for each metabolite, lysed, stored at − 80 °C for analysis. Perchloric acid (4 M) extracts were prepared, neutralized and fluorescence intensity (ATP, ex/em:535/587 nm, ADP, ex/em:535/587 nm) was monitored using an Envision Multi-label plate reader (Perkin Elmer, Waltham, MA). ATP and ADP levels were normalized to protein concentration (Bradford, BIO-RAD, Hercules, CA).

### Statistical analyses

Data represents mean ± SEM unless stated otherwise. Initial rates were analyzed using a least-squares analysis. Comparisons were made by ANOVA with Dunnett’s post-hoc analysis or one-or two-way students’ t-test using GraphPad Prism. Minimal statistical significance was set at *p* < 0.05.

## Supplementary Information

Supplementary Information.

## Data Availability

All data generated or analyzed during this study are included in this published article (and its Supplementary information files) or are available from the corresponding author.
