# Supplementary material for: Small molecule-mediated allosteric activation of the base excision repair enzyme 8-oxoguanine DNA glycosylase and its impact on mitochondrial function
Source: Sci Rep. 2022 Aug 29;12:14685. doi: 10.1038/s41598-022-18878-2 (PMC9424235; doi:10.1038/s41598-022-18878-2)
Supplement: Supplementary file 1 — Supplementary Information. [file 41598_2022_18878_MOESM1_ESM.doc]

**MATERIAL AND METHODS**

*Quantitative cell-based image acquisition and metabolite analysis.* A549 cells were imaged using the Operetta Imaging platform (Perkin Elmer; Waltham, MA) with Harmony v3.5.2 software using four fields per well, a 20X long-working distance objective (wide-field mode). Dyes were added to untreated cells (controls) to adjust for background staining and to assess dye interference from each color overlay. Cells intersecting the border were excluded from data analysis. Spatial resolution was typically 40 μm.

Determination of ROS was made by replacing cell medium with warm phosphate buffered saline (PBS plus 0.9 mM Ca^2+^, 0.5 mM Mg^2+^) and CM-H2DCFDA (DCF, 5 μM, ex/em, 460-490/500-550 nm, Life Technologies, Carlsbad, CA). This medium was replaced with Ham’s F12 plus Hoechst 33342 (5 μg/ml, ex/em:360-400/410-480 nm) and MitoTracker^TM^ orange (250 nM, ex/em:520-550/560-630 nm, Life Technologies, Carlsbad, CA). Images were segmented and the mean DCF fluorescence intensity was quantified for the entire cell region. For mitochondrial ROS, MitoSOX^TM^ Red Mitochondrial Superoxide Indicator (ex/em:520-550/560-630 nm, Life Technologies, Carlsbad, CA) was used according to manufacturer’s directions with Hoechst (5 μg/ml) and CellMask^TM^ Deep Red (5 μg/ml, ex/em:620-640/650-760 nm) for detection of the nuclear and plasma membranes, respectively. After 10 min, medium was replaced with live cell imaging solution. Fluorescence intensity was quantified within the cytoplasm/cell number.

Phosphorylated-histone 2-AX (γ-H2AX) was determined using a commercially available kit (Cellomics, Thermo Scientific, Rockford, IL) after fixation of the cells with 2% paraformaldehyde, permeabilization (0.1% Triton X-100 in PBS) and washing (PBS plus 0.3% bovine serum albumin, BSA, and 2% FBS). To detect nuclear DNA strand breaks, mouse-anti-γ-H2AX or mouse IgG was added, incubated (1 h at 22 ^o^C) and washed with PBS (plus 0.1% Tween-20). Diluted labeled goat anti-mouseIgM-DyLight^TM^ 550 (1:500 plus 0.3% BSA, ex/em:520-550/560-630 nm, Thermo Scientific, Rockford, IL) and Hoechst 33342 (5 μg/ml) were incubated (45 min at 22 ^o^C) and images of nuclear γ-H2AX were obtained. After similar cell fixation, NF-κB translocation in response to PQ was quantified using a polyclonal antibody against NF-κB (PA5-16545; 5 μg/mL) and conjugated with anti-rabbit IgG-AlexaFluor^TM^ 488 (ex/em:460-490/500-550 nm). As noted above, Hoechst 33342 (5 μg/ml) and CellMask^TM^ Deep Red were used for the detection of nuclear and plasma membranes. Texture analysis was used to determine mitochondrial membrane integrity together with MitoTracker^TM^ orange and the smart learning features of PhenoLOGIC enabled Harmony software (Perkin Elmer, Waltham, MA). MitoTracker® orange appears punctate in images of healthy mitochondria, however, in dysfunctional ones, the dye is diffuse. The phenotypes are recognized, and intensity quantified as the mean for each region. Cytochrome *c* translocation was measured using a commercially available kit (Cellomics, Thermo Scientific, Rockford, IL). Following fixation and permeabilization described above, mouse-anti-cytochrome *c* (2.5 μg/ml, Thermo Scientific, Rockford, IL) or IgG was incubated with the cells, cleared, and incubated with labeled goat-anti-mouse IgG-DyLight^TM^ 550 (1:500, with 0.3% BSA, Thermo Scientific, Rockford, IL), Hoechst 33342 (5 μg/ml) and CellMask^TM^ Deep Red (5 μg/ml). Fluorescence intensity was computed in nuclear and cytoplasmic regions. Translocase outer membrane (TOM20) immunofluorescence was similarly measured [73] using rabbit anti-TOM20 (4 μg/ml, Santa Cruz, Dallas, TX) or IgG incubated with the cells overnight (4 ^o^C) and washed. Labeled goat-anti-rabbit IgG (H&L)-Alexa Fluor^TM^ 647 (1:500, ex/em:620-640/650-700 nm, Life Technologies, Carlsbad, CA), 3% BSA and Hoechst 33342 (5 μg/ml) were added to the cells for imaging of cytoplasmic fluorescence intensity. Dynamin related protein-1 (DRP1) was detected using manufacturer’s instructions (Abcam, Cambridge, MA) after fixation and permeabilization. Rabbit-anti-DRP1 (2 mg/ml, Thermo Scientific, Rockford, IL) or IgG was incubated overnight (4 ^o^C). After washing the cells, labeled goat-anti-rabbit IgG (H&L)-Alexa Fluor^TM^ 488 and Hoechst 33342 (5 μg/ml) were incubated for 1 h (22 ^o^C). Processing was similar to TOM20. Mitofusin-1 (MFN1) was measured according to kit directions (Abcam, Cambridge, MA). Following fixation, permeabilization, addition and removal of blocking buffer (5% horse serum, Life Technologies, Carlsbad, CA), chicken-anti-MFN1 or chicken-IgG (10 μg/ml) was incubated overnight (4^o^C) with the cells. Labeled goat-anti-chicken IgG (H&L)-Alexa Fluor^TM^ 488 (1:250, Life Technologies, Carlsbad, CA) plus Hoechst 33342 (5 μg/ml) were incubated (1 h) with the cells and processed as described above. DNA ligase III was quantified as described previously [74] in fixed and cells were processed as above. Polyclonal rabbit anti-DNA Ligase III antibody (5 mg/ml, Novus Biological, Littleton, CO) or IgG were incubated overnight with cells (4 ^o^C). After washing, the cells were incubated (45 min) at room temperature with labeled goat-anti-rabbit IgG (H&L)-DyLight^TM^ 550 (1:500, Thermo Scientific, Rockford, IL) and Hoechst 33342 (5 μg/ml). CellMask^TM^ Deep Red (5 μg/ml) was briefly added. Fluorescence from DNA Ligase III-DyLight^TM^ 550 was quantified in the cytoplasm. For OGG1 content, the cell plates were fixed in 4% paraformaldehyde and permeabilized (0.1% Triton X-100 in PBS) for 15 min at room temperature. Non-specific binding sites were blocked with 10% goat serum (Life Technologies; Carlsbad, CA), 1% BSA, and 0.1% Tween-20 in PBS and 2 h incubation at room temperature. The buffer was removed and replaced with detection buffer (5% BSA, 0.1% Tween-20, 2.7 μg/ml rabbit anti-OGG1 antibody (Cambridge Research Biologicals; Cleveland, UK) and incubated overnight at room temperature.  Labeled goat-anti-rabbit IgG (H &L)-Alexa Fluor^TM^ 488 (Life Technologies; Carlsbad, CA) plus Hoechst 33342 (5 μg/ml) were incubated (1 h) with the cells and processed as described above.

For glutathione content, cells were treated with 5% sulfosalicylic acid, lysed by freeze-thaw and spun down. Supernatants were utilized for determination of glutathione content using a commercially available kit (Arbor Assays, Atlanta, GA).

**SUPPLEMENTARY DATA**

Supplementary Figure S1.


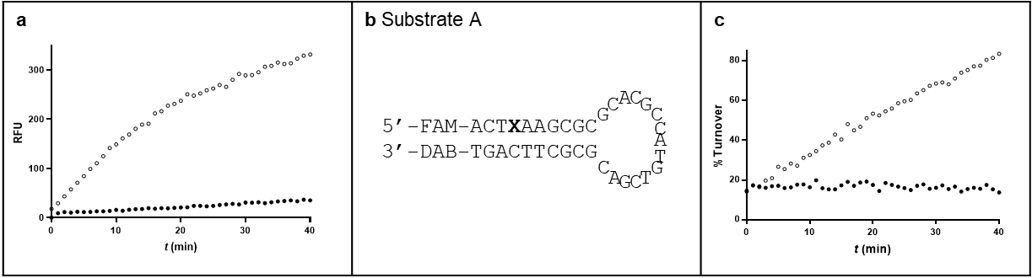


Supplementary Figure S1. Kinetic assay substrate and typical example of reaction rate. (**a**) Progress curve of OGG1 reaction. The time course of an OGG1 reaction with 100 nM of a DNA substrate from an earlier design containing five base pairs 5' to 8-oxo-dG in the stem of the hairpin DNA substrate and 250 μM 8-bromoGua in the presence (○) or absence (●) of 10 nM recombinant human OGG1. The Y-axis represents relative fluorescence units (RFU). (**b**) Predicted secondary structure of the 8-oxo-dG containing hairpin substrate (Substrate A). The substrate contains a 10-base pair stem and a 15-base loop. The 8-oxo-dG residue is designated X. (**c**) Progress curve of OGG1 reaction. The time course of an OGG1 reaction with 100 nM Substrate A and 200 μM 8-bromoGua in the presence (○) or absence (●) of 10 nM recombinant human OGG1. Cleavage of Substrate A at 8-oxo-dG allows the 5' FAM-labeled triplet DNA oligo to dissociate which releases the quench effect of DAB to increase fluorescence emission.

Supplementary Figure S2.


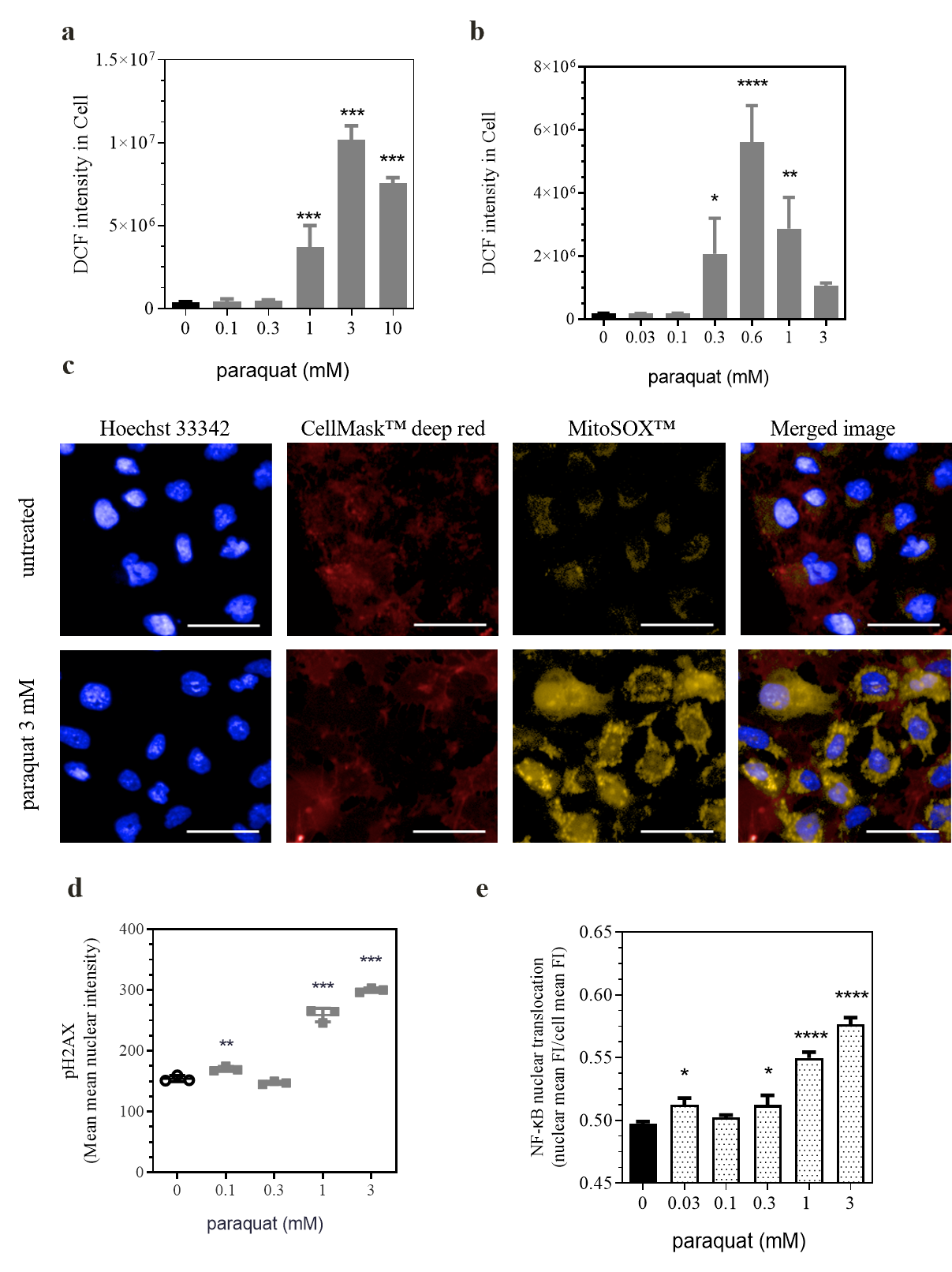


Supplementary Figure S2. Effects of paraquat on reactive oxygen species production, nuclear DNA damage and NF-κB translocation. In (**a**) and (**b**), A549 cells were treated with paraquat for 24 and 48 h, respectively, and images were obtained using CM-H2DCFDA (DCF) followed by identification of mitochondria using MitoTracker^TM^ orange and a 20X magnification (4 fields per well in triplicate). Fluorescence intensity of DCF within the cell was averaged based on the number of imaged cells. The paraquat EC_50_ value was 1.6 mM with a maximum of 30-fold over baseline using 3 mM at 24 h. By 48 h, the EC_50_ value was 0.34 mM, thereby shifting the concentration-response curve to the left and reaching a maximum of 30-fold at 0.6 mM. The graphical representations are the average of three experiments. In (**c**), exemplar images of mitochondrial ROS using MitoSOX^TM^ (overlaid orange), Hoechst (overlaid blue) and CellMask^TM^ deep red (overlaid red) are shown in the absence and presence of 3 mM paraquat from one experiment in triplicate. Panel (**d**) shows nuclear DNA damage measuring γH2AX in A549 cells exposed to varying levels of paraquat for 48 h. A 2-fold rise resulted from the highest paraquat concentration. At 24 h post-paraquat, essentially no changes in this parameter occurred. Values represent the average of triplicate measures from one independent experiment. Shown in panel (**e**) is NF-κB translocation which was quantified from images obtained from A549 cells as described in Methods. The concentration-dependent increase was obtained after a 24 h exposure to paraquat. Values represent one independent experiment in triplicate. Statistical analyses were conducted using ANOVA with Dunnett’s post-test. Asterisks represent from left to the right side of the panels in: (**a**) *p* < 0.0001, (**b**) 0.0291, < 0.0001 and 0.0022, (**d**) 0.0038, <0.0001 and < 0.0001, and (**e**) 0.0418, 0.0476, < 0.0001 and <0.0001, respectively.

Supplementary Figure S3.


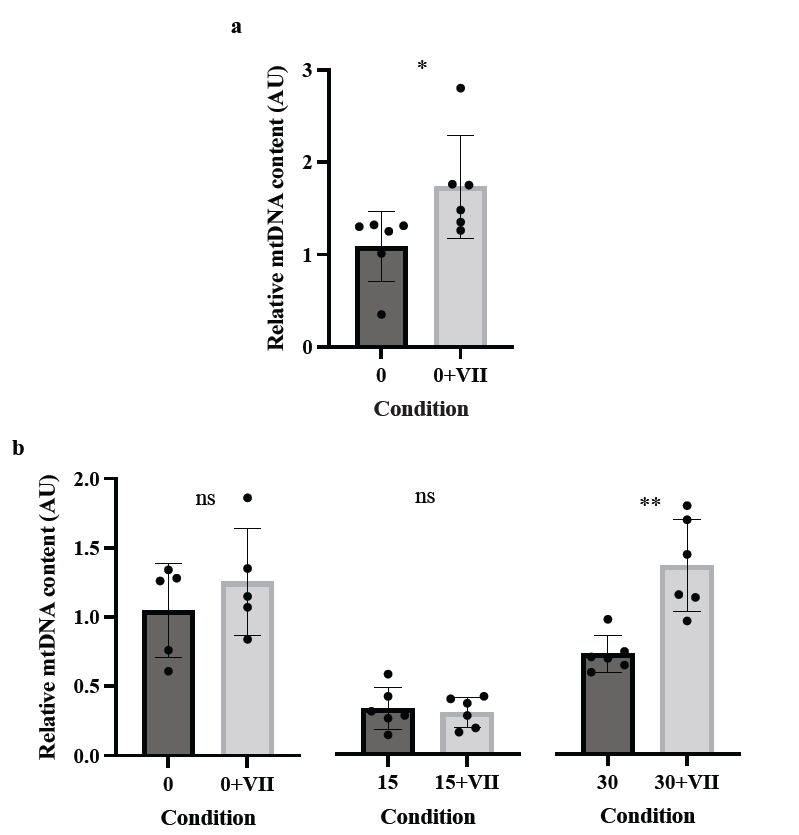


Supplementary Figure S3. Effect of hydrogen peroxide on mtDNA content and recovery with and without Compound **VII**. MEFs were pre-incubated with compound **VII** (25 μM) for four h prior to challenge with H_2_O_2_ (400 μM). Samples were collected under basal conditions (**a**) and at time zero, 15 and 30 min of recovery (**b**) for determination of mtDNA content as described in Methods. Statistical comparisons were made using unpaired Student’s t-test with Welch’s correction (* *p* = 0.0458, ***p* = 0.0039, respectively).

Supplementary Figure S4.


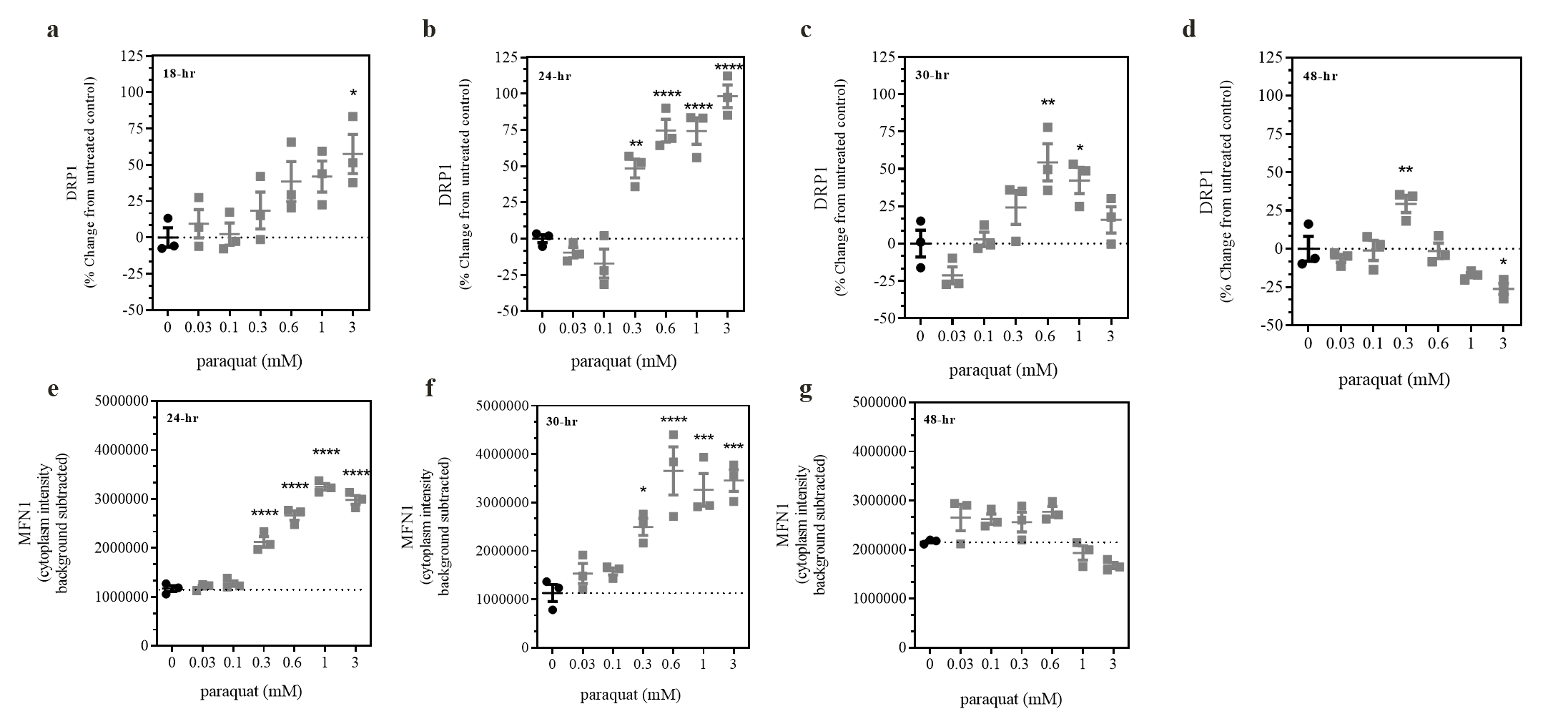


Supplementary Figure S4. Time course of paraquat-induced changes in DRP1 and MFN1. In panels (**a**-**d**), samples images were quantified at 18, 24, 30 and 48 h post-paraquat application to A549 cells. Data are presented as the percent change from baseline which is depicted by the dotted line. Changes in MFN1 content are provided in panels (**e**-**g**) for time periods of 24, 30 and 48 h of paraquat treatment. The data indicate that DRP1 and MFN1 content rise early after paraquat exposure but diminish over time. Values represent the average of triplicate measures from a single independent experiment. Statistical analyses were conducted using ANOVA with Dunnett’s post-test. Significance values denoted by the asterisks from left to right side of the panels are: (**a**) *p* = 0.0114, (**b**) *p* = 0.0017, < 0.0001, < 0.0001, < 0.0001, (**c**) *p* = 0.0041, 0.0248, (**d**) *p* = 0.0063, 0.014, (**e**) *p* < 0.0001, 0.0001, 0.0001, 0.0001, and (**f**) *p* = 0.0154, < 0.0001, 0.0004, 0.0002, respectively.

Supplementary Figure S5.


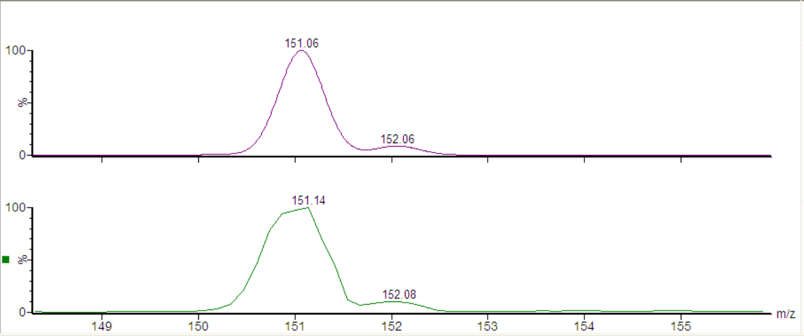


Supplemental Figure S5. Mass spectrum of the sample evaluated in the OGG1 activation experiment expanded to show the m/z range expected for the mass of 9-deazaGua. The mass spectrum of 9-deazaGua (bottom panel) was determined using a 10 mM DMSO solution. The spectrum displays a major peak at m/z 151 and a minor peak at m/z 152, consistent with [M1+H]+ and [M2+H]+ ions for the [^14^N]9-deazaGua and [^15^N]9-deazaGua species, respectively, present in the sample tested. A theoretical spectrum of 9-deazaG assuming a 0.4% natural abundance of the [^15^N]9-deazaGua is given by the top panel. The theoretical and experimental spectra were superimposable, indicating that the purity of the 9-deazaGua sample was close to 100%.

Supplementary Table S1.


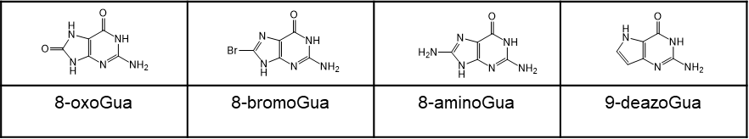


Supplemental Table S1. Chemical structures of 8-oxoGua and its analogs 8-bromoGua, 8-aminoGua and 9-deazaGua.

Supplementary Table S2.


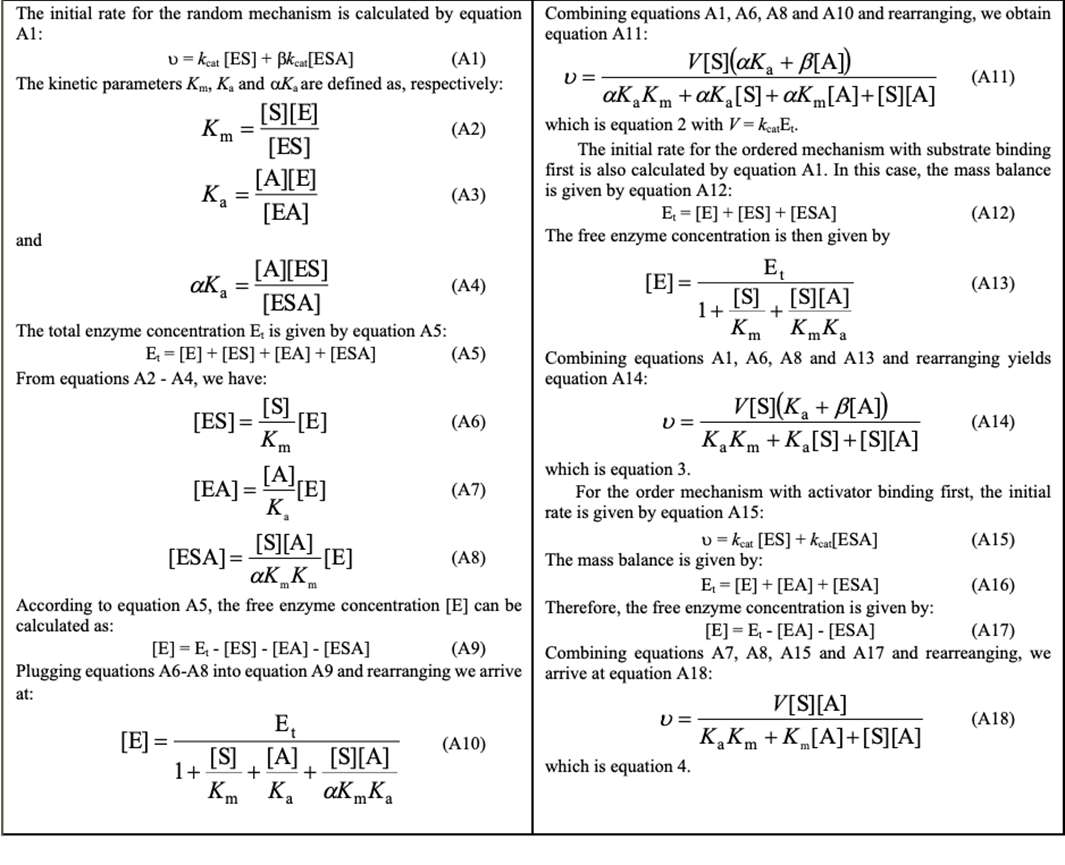


Supplemental Table S2. List of equations used for derivation of equation 4.

Supplementary Table S3.


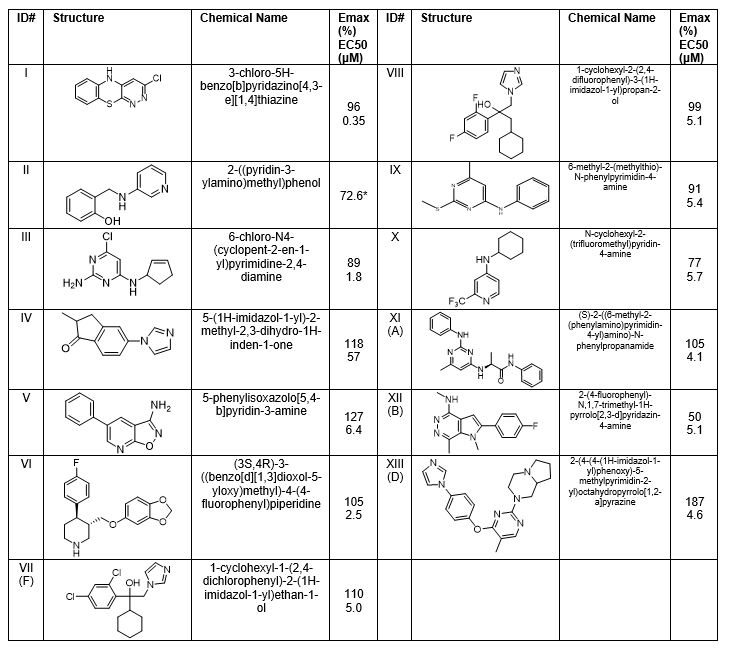


Supplemental Table S3. Small molecule OGG1 activators. Purity ranged from 93-100%. Pharmacological values were obtained with the fluorescent kinetic assay described in Methods. E_max_ data is represented as percent activation relative to the mean activation obtained with a saturating concentration (500 μM) of 8-bromoGua (set as 100%) for the plate of compounds tested. *For compound **II**, only percent activation at 10 μM is provided as E_max_ and EC_50_ values were not determined. Compounds **VII**, **XI**, **XII**, and **XIII** are also referred to as F, A, B, and D respectively, as described previously [27]. Surface plasmon resonance (SPR) was determined for compounds **VII**, **VII**, **IX**, **X**, **XI**, and **XII**, and the pXC_50_ values were: 5.2, 5.3, 5.4, 5.7, 5.6 and 6.4, respectively. SPR measurements of specific binding were determined as described Methods and Materials.
